# Supplementary material for: MGAT1-Guided complex N-Glycans on CD73 regulate immune evasion in triple-negative breast cancer
Source: Nat Commun. 2025 Apr 15;16:3552. doi: 10.1038/s41467-025-58524-9 (PMC11997035; doi:10.1038/s41467-025-58524-9)
Supplement: Supplementary file 1 — Supplementary Information [file 41467_2025_58524_MOESM1_ESM.pdf]

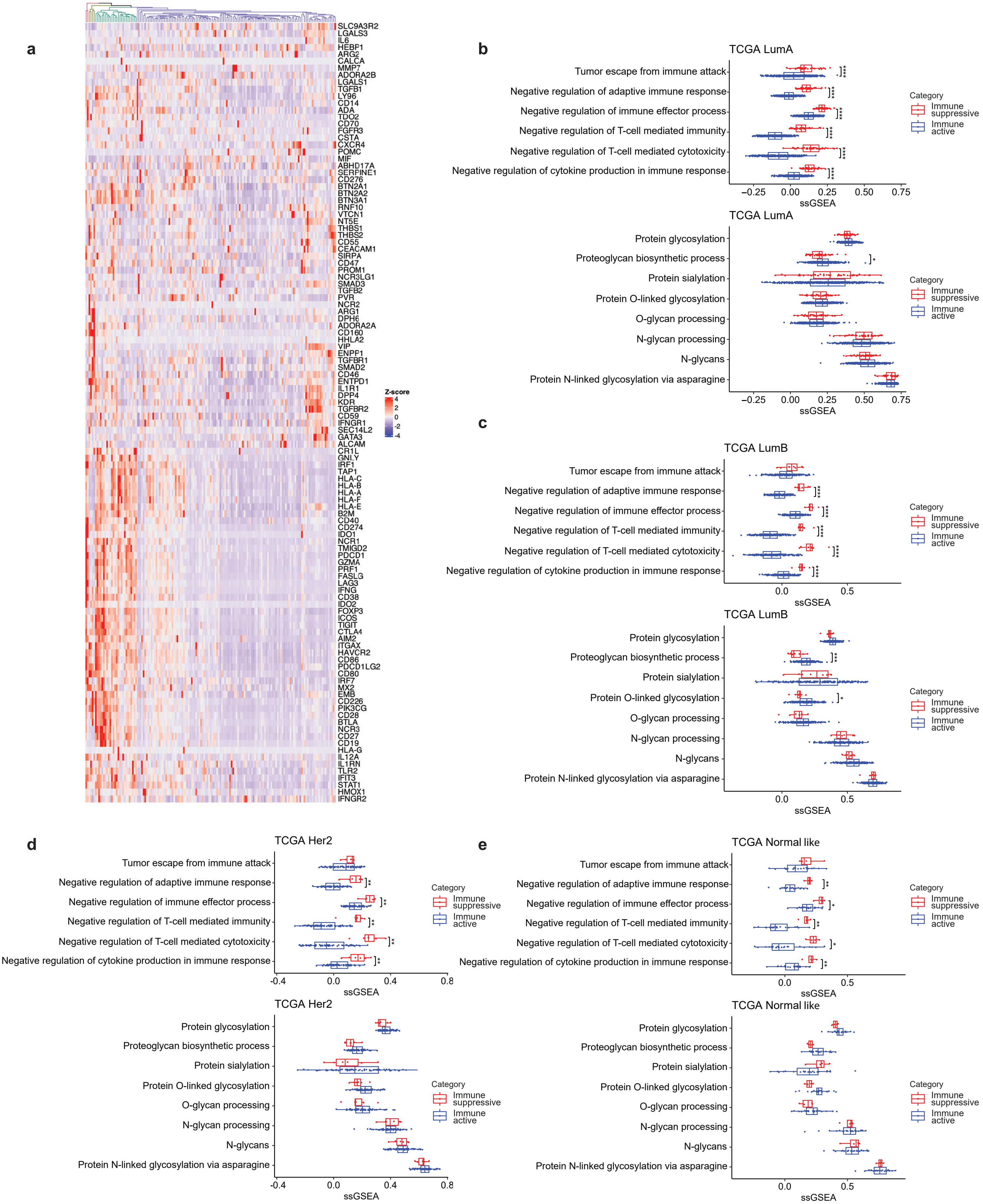
a
SLC9A3R2
LGALS3
IL6
HEBP1
ARG2
CALCA
MMP7
ADORA2B
LGALS1
TGFB1
LY96
CD14
ADA
TDO2
CD70
FGFR3
CSTA
CXCR4
POMC
MIF
ABHD17A
SERPINE1
CD276
BTN2A1
BTN2A2
BTN3A1
RNF10
VTCN1
NT5E
THBS1
THBS2
CD55
CEACAM1
SIRPA
CD47
PROM1
NCR3LG1
SMAD3
TGFB2
PVR
NCR2
ARG1
DPH6
ADORA2A
CD160
HHLA2
VIP
ENPP1
TGFBR1
SMAD2
CD46
ENTPD1
IL1R1
DPP4
KDR
TGFBR2
CD59
IFNGR1
SEC14L2
GATA3
ALCAM
CR1L
GNLY
IRF1
TAP1
HLA-C
HLA-B
HLA-A
HLA-F
HLA-E
B2M
CD40
CD274
IDO1
NCR1
TMIGD2
PDCD1
GZMA
PRF1
FASLG
LAG3
IFNG
CD38
IDO2
FOXP3
ICOS
TIGIT
CTLA4
AIM2
ITGAX
HAVCR2
CD86
PDCD1LG2
CD80
IRF7
MX2
EMB
CD226
PIK3CG
CD28
BTLA
NCR3
CD27
CD19
HLA-G
IL12A
IL1RN
TLR2
IFIT3
STAT1
HMOX1
IFNGR2
Z-score
4
2
0
-2
-4
b
TCGA LumA
Tumor escape from immune attack
Negative regulation of adaptive immune response
Negative regulation of immune effector process
Negative regulation of T-cell mediated immunity
Negative regulation of T-cell mediated cytotoxicity
Negative regulation of cytokine production in immune response
−0.25
0.00
0.25
0.50
0.75
ssGSEA
Category
Immune suppressive
Immune active
TCGA LumA
Protein glycosylation
Proteoglycan biosynthetic process
Protein sialylation
Protein O-linked glycosylation
O-glycan processing
N-glycan processing
N-glycans
Protein N-linked glycosylation via asparagine
−0.25
0.00
0.25
0.50
0.75
ssGSEA
Category
Immune suppressive
Immune active
c
TCGA LumB
Tumor escape from immune attack
Negative regulation of adaptive immune response
Negative regulation of immune effector process
Negative regulation of T-cell mediated immunity
Negative regulation of T-cell mediated cytotoxicity
Negative regulation of cytokine production in immune response
0.0
0.5
ssGSEA
Category
Immune suppressive
Immune active
TCGA LumB
Protein glycosylation
Proteoglycan biosynthetic process
Protein sialylation
Protein O-linked glycosylation
O-glycan processing
N-glycan processing
N-glycans
Protein N-linked glycosylation via asparagine
0.0
0.5
ssGSEA
Category
Immune suppressive
Immune active
d
TCGA Her2
Tumor escape from immune attack
Negative regulation of adaptive immune response
Negative regulation of immune effector process
Negative regulation of T-cell mediated immunity
Negative regulation of T-cell mediated cytotoxicity
Negative regulation of cytokine production in immune response
-0.4
0.0
0.4
0.8
ssGSEA
Category
Immune suppressive
Immune active
TCGA Her2
Protein glycosylation
Proteoglycan biosynthetic process
Protein sialylation
Protein O-linked glycosylation
O-glycan processing
N-glycan processing
N-glycans
Protein N-linked glycosylation via asparagine
-0.4
0.0
0.4
0.8
ssGSEA
Category
Immune suppressive
Immune active
e
TCGA Normal like
Tumor escape from immune attack
Negative regulation of adaptive immune response
Negative regulation of immune effector process
Negative regulation of T-cell mediated immunity
Negative regulation of T-cell mediated cytotoxicity
Negative regulation of cytokine production in immune response
0.0
0.5
ssGSEA
Category
Immune suppressive
Immune active
TCGA Normal like
Protein glycosylation
Proteoglycan biosynthetic process
Protein sialylation
Protein O-linked glycosylation
O-glycan processing
N-glycan processing
N-glycans
Protein N-linked glycosylation via asparagine
0.0
0.5
ssGSEA
Category
Immune suppressive
Immune active

Supplementary Fig. 1: The N-glycosylation signaling pathway is hyperactivated in TNBC but not other breast cancer subtypes. a Heatmap showing hierarchical clustering of row-scaled breast cancer gene expression TPM data from TCGA of subset of immune related genes differentially expressed in four breast cancer types. Each column represents a sample; each row represents a protein. **b-e** Distribution of single sample GSEA scores of LumA (**b**) (immune suppressive subgroup, n=42; immune active subgroup, n=438), LumB (**c**) (immune suppressive subgroup, n=8; immune active subgroup, n=189), Her2+ (**d**) (immune suppressive subgroup, n=6; immune active subgroup, n=68), and Normal-like (**e**) (immune suppressive subgroup, n=4; immune active subgroup, n=23) separated by immune subgroup, using gene sets involved in immune functions and glycosylation. P-values are generated from two-sided Wilcoxon tests and included in Source Data. *p<0.05, **p<0.01, ***p<0.001, and ****p<0.0001. Source data are provided as a Source Data file.

a

Full N-glycan biosynthesis gene list (row) and immune related gene list (column) used for Pearson correlation analysis in Fig. 1d in order

Row (1-23)	Row (24-46)	Colum (1-23)	24-46	47-69	70-92	93-112
MGAT1	ALG6	BTN2A1	ADORA2A	SMAD3	IFNG	ADA
ST6GAL1	DDOST	PVR	B2M	CD274	PDCD1	CD27
MGAT4A	DOLPP1	CD80	CD55	FOXP3	TGFB2	GNLY
MAN2A1	ALG1	RNF10	CR1L	NCR1	CTLA4	IL6
MGAT2	RPN2	ADORA2B	HAVCR2	PDCD1LG2	IRF7	LY96
ALG2	DPAGT1	BTN2A2	NCR3LG1	POMC	ALCAM	TDO2
MAN1A2	ALG8	BTN3A1	TAP1	PRF1	MIF	TGFBR2
ALG11	MGAT5B	CD276	CD46	AIM2	TGFB1	IDO1
MAN1C1	ALG5	TGFBR1	CXCR4	EMB	TIGIT	IL1RN
MAN1A1	TUSC3	VTCN1	FGFR3	FASLG	TMIGD2	VIP
ALG13	STT3A	CEACAM1	HMOX1	HEBP1	BTLA	CD19
ALG9	MAN1B1	STAT1	SMAD2	HLA-A	CD226	CD40
MGAT4B	DPM1	THBS2	CD38	HLA-G	HLA-F	CALCA
MGAT3	B4GALT3	IFIT3	CD47	ICOS	NCR2	CD14
FUT8	RPN1	CD59	HLA-B	IL12A	SEC14L2	CD70
MAN2A2	GANAB	IFNGR2	HLA-C	IL1R1	ARG1	SLC9A3R2
ALG10B	MOGS	SERPINE1	IDO2	ITGAX	DPP4	LGALS1
ALG10	DAD1	ARG2	NT5E	LAG3	GZMA	GATA3
MGAT5	ALG3	CD86	ENTPD1	MX2	HHLA2	LGALS3
ALG12	DPM2	PROM1	IFNGR1	CD160	HLA-E	CSTA
STT3B	ALG14	SIRPA	KDR	CD28	IRF1	
B4GALT1	DPM3	THBS1	MMP7	DPH6	NCR3	
RFT1	B4GALT2	TLR2	PIK3CG	ENPP1	ABHD17A	

b

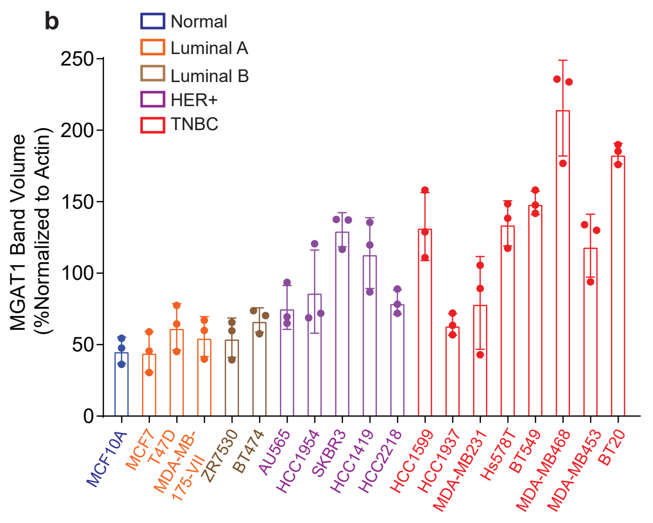

c

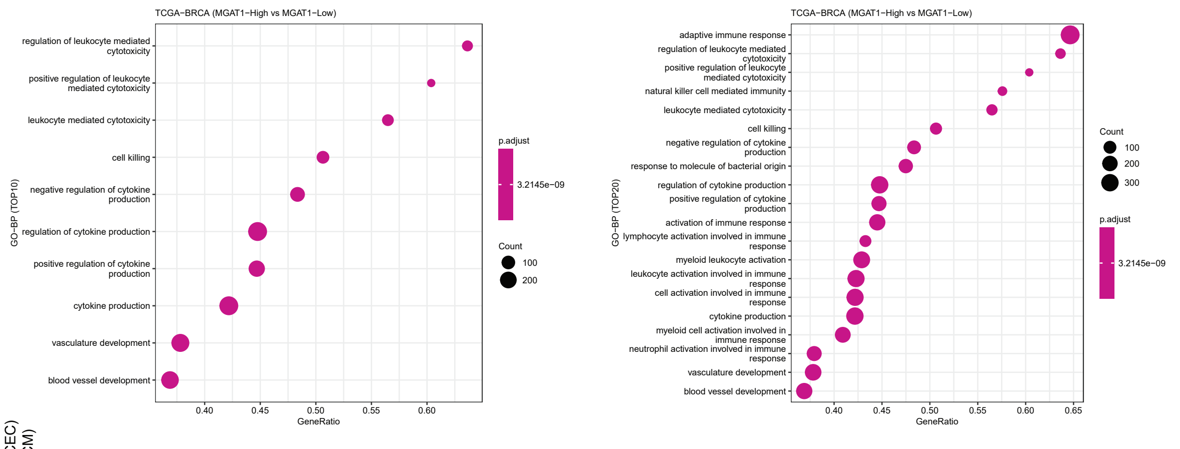

f **d** **e** **g**

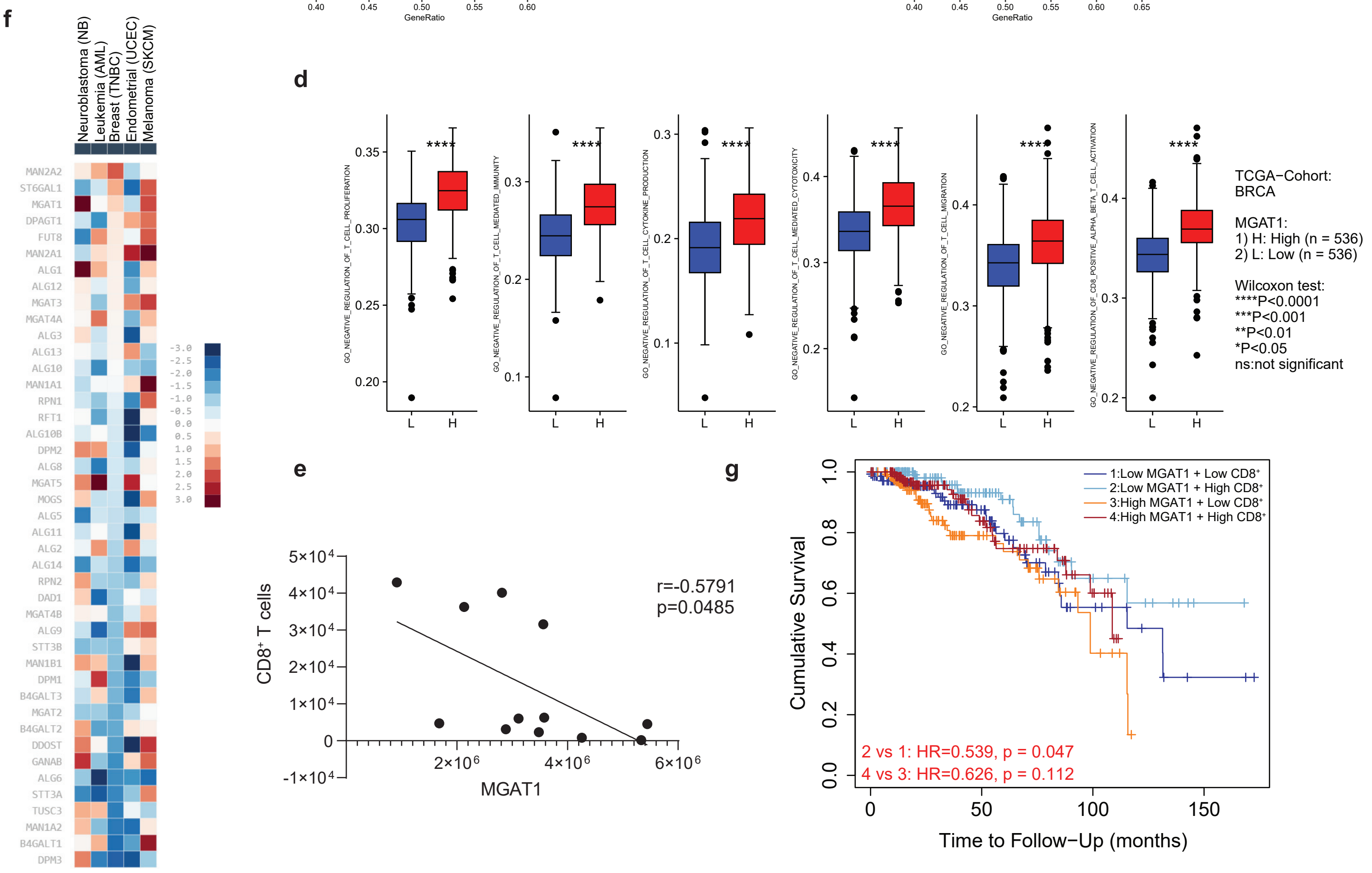

Supplementary Fig. 2: Accumulation of MGAT1, a glycosyltransferase, is dramatically associated with an unfavorable tumor immune response and prognosis in immune-cold breast cancers. a Full N-glycan biosynthesis gene list (row) and immune related gene list (column) used for Pearson correlation analysis in Fig. 1d in order. **b** The immunoblots of MGAT1 in normal human mammary epithelial cells and various subtypes of breast cancer cells were quantified with Image Lab and normalized to actin. **c, d** Go-BP pathway analysis using TCGA dataset identifying the most affected pathways between MGAT1 high vs MGAT1 low breast cancer group. The MGAT1 high population presents increased negative regulation on T cell-mediated immune response. **e** The analysis of MGAT1 protein expression and $CD8^+$ T cell on breast cancer specimens TMA (n=12) using QuPath showing the negative correlation between MGAT1 expression with $CD8^+$ T cell. **f** The T-cell dysfunction scores of N glycan biosynthesis genes generated by TIDE with core databases and the gene list was ranked based on correlation to TNBC. N-glycan biosynthesis genes were collected from KEGG_N_GLYCAN_ BIOSYNTHESIS (hsa00510). **g** Kaplan-Meier curves of TNBC patients using multivariable Cox proportional hazard models for the corresponding $CD8^+$ T cell and MGAT1 levels. High MGAT1 expression diminished the therapeutic benefit created by high $CD8^+$ T cell infiltration. P-values are from Wilcoxon tests. ****p<0.0001. Source data are provided as a Source Data file.

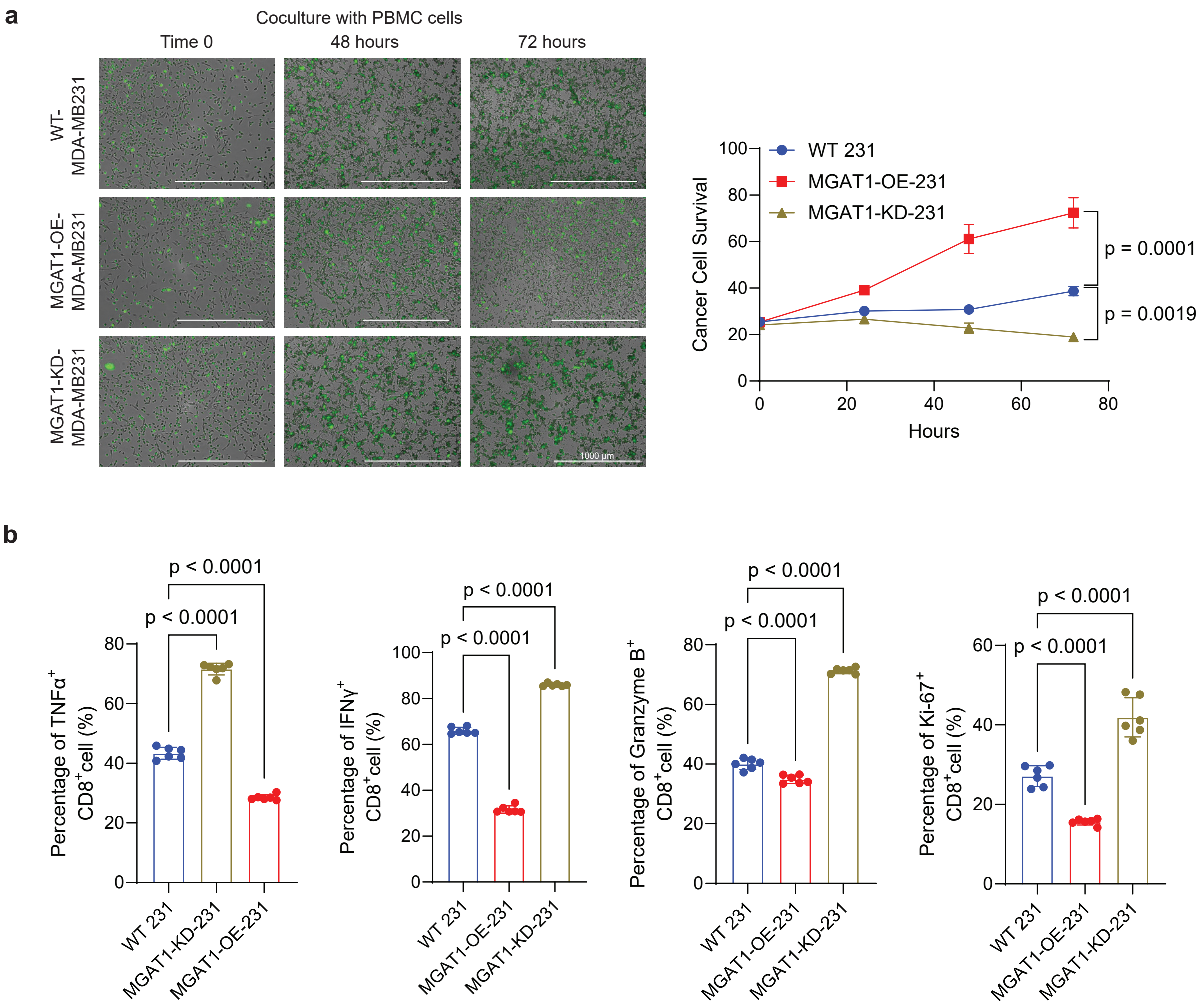

Supplementary Fig. 3: Elevated expression of MGAT1 inhibits tumor immune response in coculture systems. a The survival of MDA-MB231, MDA-MB231-MGAT1-OE, and MDA-MB231-MGAT1-KD cells (green) in 2D coculture with pre-activated PBMCs was monitored by time lapse image-based quantification. The data are presented as mean ± SEM from three replicates from a representative experiment. **b** MDA-MB231, MDA-MB231-MGAT1-OE, and MDA-MB231-MGAT1-KD cells were cocultured with pre-activated human PBMCs, and IFNγ^{+}, TNFα^{+}, Granzyme B^{+}, Ki-67^{+} CD8^{+} T cell populations were measured and quantified using flow cytometry. Data (represented as means ± SEM), images and flow cytometry are representative of three independent experiments. Statistical significance was determined using two-way ANOVAs followed by Tukey's multiple comparison tests (**a**) or one-way ANOVA with Tukey's multiple comparisons test (**b**). Source data are provided as a Source Data file.

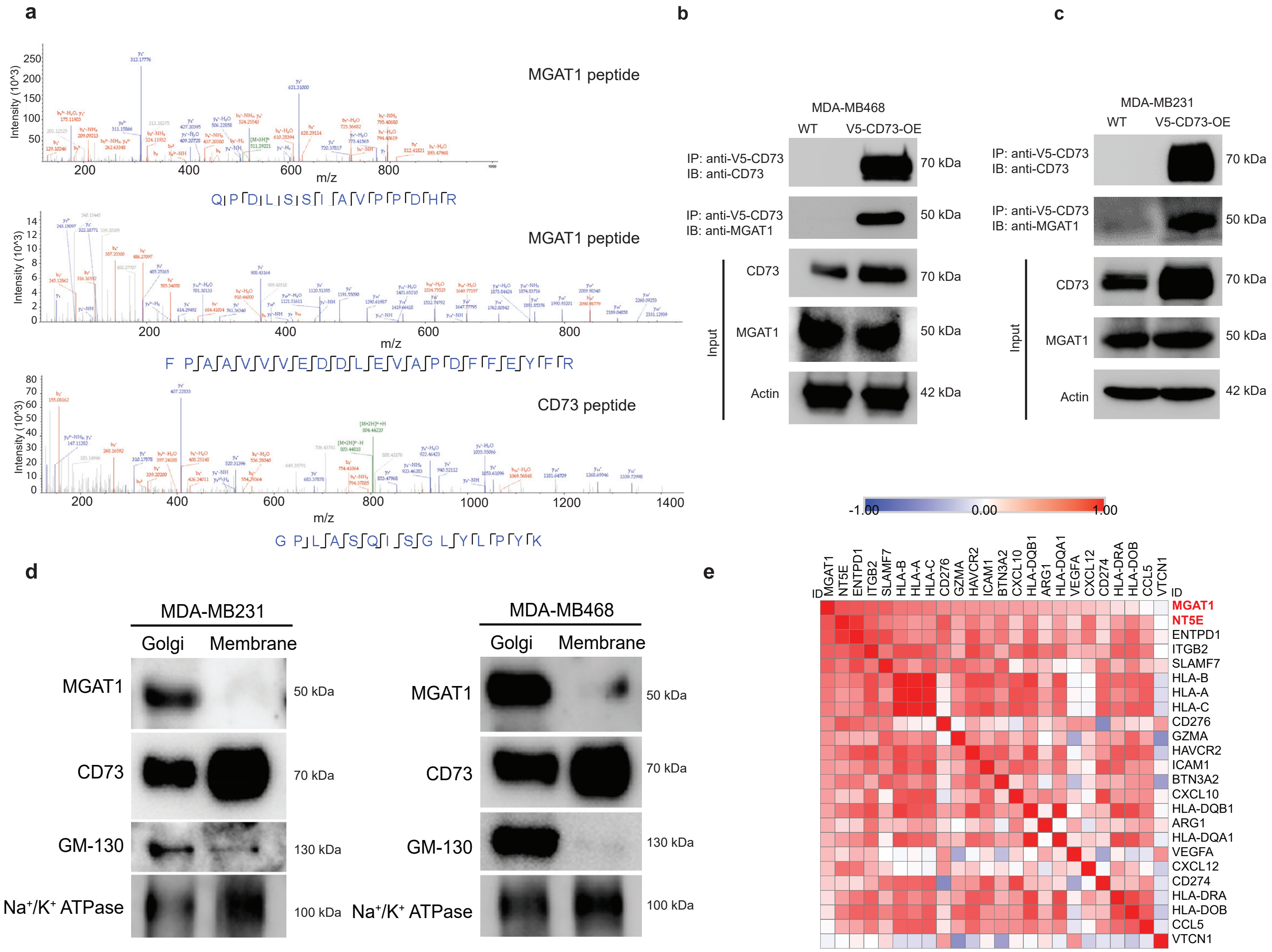

Supplementary Fig. 4: Investigation of the biochemical interaction between MGAT1 and CD73. a Representative peptide of CD73 and MGAT1 discovered by mass spectrometry. **b, c** The biochemical interaction between MGAT1 and CD73 in MDA-MB468 cells was validated by coimmunoprecipitation of ectopic V5-CD73. The samples derived from the same experiment but different gels for CD73 and MGAT1, and β-ACTIN were processed in parallel. **d** The colocalization of MGAT1 and CD73 in the Golgi was validated by fractionation of the Golgi and membrane sections of the cells followed by immunoblot. The samples derived from the same experiment but different gels for GM-130, Na^+/K^+ ATPase and CD73 and MGAT1, and β-ACTIN were processed in parallel. **e** Spearman's rank correlation analysis shows that MGAT1 protein expression is highly positively correlated with several immune regulators, and CD73 (gene name: NT5E) is the most positively correlated one. Data (mean ± SEM) and western blot are representative of three independent experiments. Source data are provided as a Source Data file.

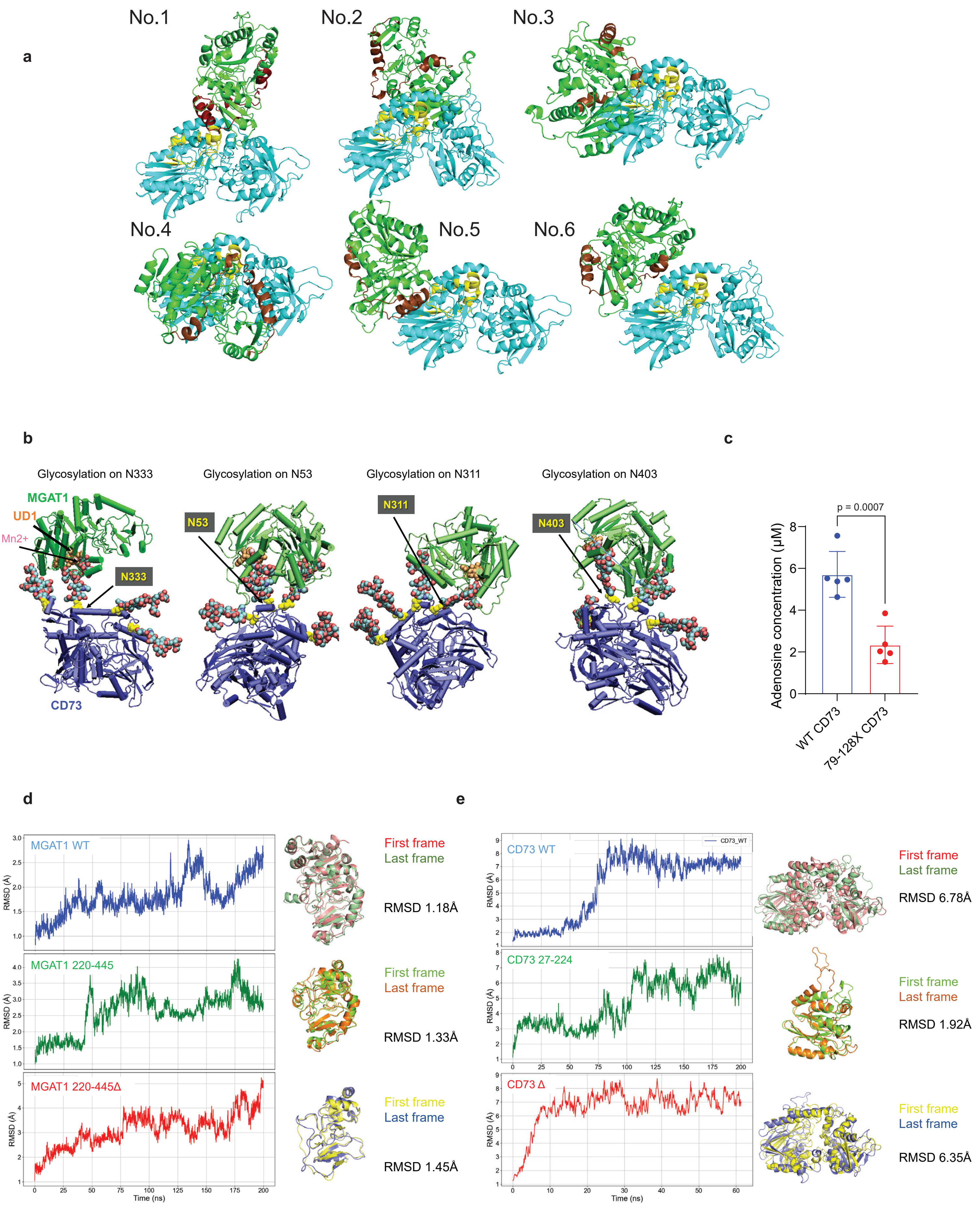

a
No.1
No.2
No.3
No.4
No.5
No.6
b
Glycosylation on N333
Glycosylation on N53
Glycosylation on N311
Glycosylation on N403
MGAT1
UD1
Mn2+
N333
N53
N311
N403
CD73
c
p = 0.0007
Adenosine concentration (μM)
WT CD73
79-128X CD73
d
MGAT1 WT
MGAT1 220-445
MGAT1 220-445Δ
RMSD (Å)
Time (ns)
First frame
Last frame
RMSD 1.18Å
RMSD 1.33Å
RMSD 1.45Å
e
CD73 WT
CD73_WT
CD73 27-224
CD73 Δ
RMSD 6.78Å
RMSD 1.92Å
RMSD 6.35Å

Supplementary Fig. 5: Investigation of the stability and conformation of MAGT1 and CD73 fragments with molecular simulation and alternative poses of CD73 and MGAT1 predicted by AlphaFold3. a MGAT1 structure predicted by AlphaFold3 and CD73 are docked together by ClusPro. CD73 is shown in cyan and MGAT1 in green. The interfacial residues detected by experiments (Fig. 4b, d) on MGAT1 (residue 321-370) and CD73 (residues 79-128) are highlighted in brown and yellow, respectively. No.2-6 indicates five poses of truncated CD73-MGAT1 complex, predicted fully by AlphaFold3 are shown. The experimentally detected interfacial residues show no interaction in the AlphaFold3 predicted model. **b** Docking-predicted complexes of active MGAT1 (*green*) with CD73 (*blue*) monomer carrying Man5 glycan on asparagine residues (*yellow* spheres) N53, N311, N333, and N403. Man5 glycan is shown in *red* and *cyan* spheres, which represent oxygen and carbon atoms. MGAT1-bound UD1 and Mn^{2+} are displayed in *orange* and *pink* spheres. **c** The adenosine levels were determined in MDA-MB468 cells expressing CD73 WT or CD73Δ79-128. **d** Different constructs of MGAT1 are predicted by AlphaFold3 and MD simulation was carried out to assess these predicted constructs stability. RMSD progression over time is plotted for MGAT1 WT (residue sequence from P101 to N445), MGAT1 220-445 (residue sequence from E220 to N445), and MGAT1 220-445Δ (residue sequence from E220 to N445 with the deletion from H321 to V370) α-carbon position during the 200ns MD simulation. The superposition of the first and last frame of each of these MD runs were shown for visualization of the actual structural fluctuation. **e** Similar to **d,** the following constructs of CD73 were also predicted by AlphaFold3 to assess the stability of the fragments CD73 WT (residue sequence from W27 to S549, PDB ID: 4H1S), CD73 27-224 (residue sequence from W27 to E224), and CD73 Δ (residue sequence from W27 to S549 with the deletion from L79 to I128). RMSD over time is plotted over 200ns for CD73 WT, CD73 27-224, and 70ns for CD73 Δ. Data (represented as means ± SEM) are representative of three independent experiments. Statistical significance was determined using unpaired t test (**c**). Source data are provided as a Source Data file.

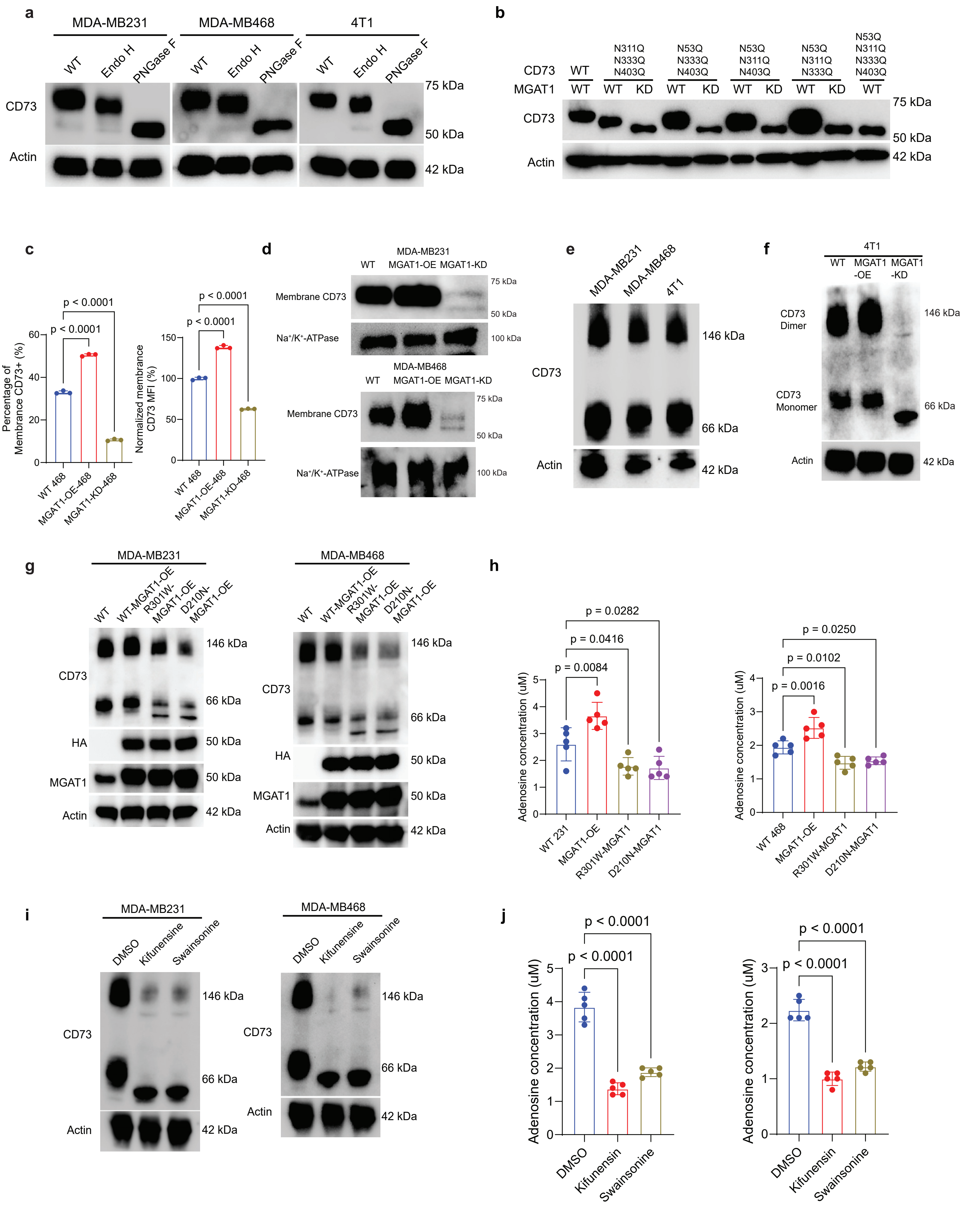

a
MDA-MB231
MDA-MB468
4T1
WT
Endo H
PNGase F
CD73
Actin
75 kDa
50 kDa
42 kDa
b
CD73
MGAT1
WT
N311Q
N333Q
N403Q
N53Q
N311Q
N53Q
N311Q
N333Q
N403Q
KD
Actin
c
Percentage of Membrance CD73+ (%)
Normalized membrane CD73 MFI (%)
p < 0.0001
WT 468
MGAT1-OE-468
MGAT1-KD-468
d
MDA-MB231
MDA-MB468
WT
MGAT1-OE
MGAT1-KD
Membrane CD73
Na+/K+-ATPase
100 kDa
e
MDA-MB231
MDA-MB468
4T1
146 kDa
66 kDa
f
CD73 Dimer
CD73 Monomer
MGAT1 -OE
MGAT1 -KD
g
WT-MGAT1-OE
R301W-MGAT1-OE
D210N-MGAT1-OE
HA
MGAT1
h
Adenosine concentration (uM)
p = 0.0282
p = 0.0416
p = 0.0084
p = 0.0250
p = 0.0102
p = 0.0016
WT 231
MGAT1-OE
R301W-MGAT1
D210N-MGAT1
WT 468
i
DMSO
Kifunensine
Swainsonine
j
p < 0.0001
DMSO
Kifunensin
Swainsonine

Supplementary Fig. 6: MGAT1-mediated glycosylation orchestrates CD73 dimerization. **a** The whole cell lysates of MDA-MB468, MDA-MB231 and 4T1 cells were treated with endoglycosidase H and PNGase F and the CD73 molecular sizes were determined by immunoblotting. **b** The protein molecular size of CD73 carried indicated triple-mutation at N-glycan sites in MGAT1-WT and KD HEK-293T cells were determined by immunoblotting. **c** The MFI of membrane CD73 was measured with flow cytometry in MGAT1-WT/OE/KD-MDA-MB468. ****$p < 0.0001$. Data (mean ± SEM) are representative of at least three independent experiments. **d** The membrane fractionated protein expression of CD73 in MGAT1-WT/OE/KD-human TNBC cell lines was determined by immunoblotting. **e** CD73 dimerization in MDA-MB231, MDA-MB468, and 4T1 cells was determined by immunoblotting with semi-native gel. **f** CD73 dimerization in 4T1 cells with MGAT1-OE/KD was determined by immunoblotting with semi-native gel. **g** CD73 dimerization in MDA-MB231 and MDA-MB468 cells with over-expression of WT-/R301W-/D210N-MGAT1 was determined by immunoblotting with semi-native gel. The samples derived from the same experiment but different gels for HA, CD73 and MGAT1, and β-ACTIN were processed in parallel. **h** Adenosine levels were determined in MDA-MB231 and MDA-MB468 cells with over-expression of WT-/R301W-/D210N-MGAT1. **i** MDA-MB231 and MDA-MB468 cells were treated with 20 μM kifunensine or swainsonine for 48 hours and the CD73 dimerization were determined by immunoblotting with semi-native gel. **j** MDA-MB231 and MDA-MB468 cells were treated with 20 μM kifunensine or swainsonine for 48 hours and the adenosine concentration in the medium was measured with Adenosine Assay from Cell Biolabs. Data (represented as means ± SEM), western blots and flow cytometry are representative of three independent experiments. Statistical significance was determined using one-way ANOVA with Tukey's multiple comparisons test (**c**, **h**, **j**). Source data are provided as a Source Data file.

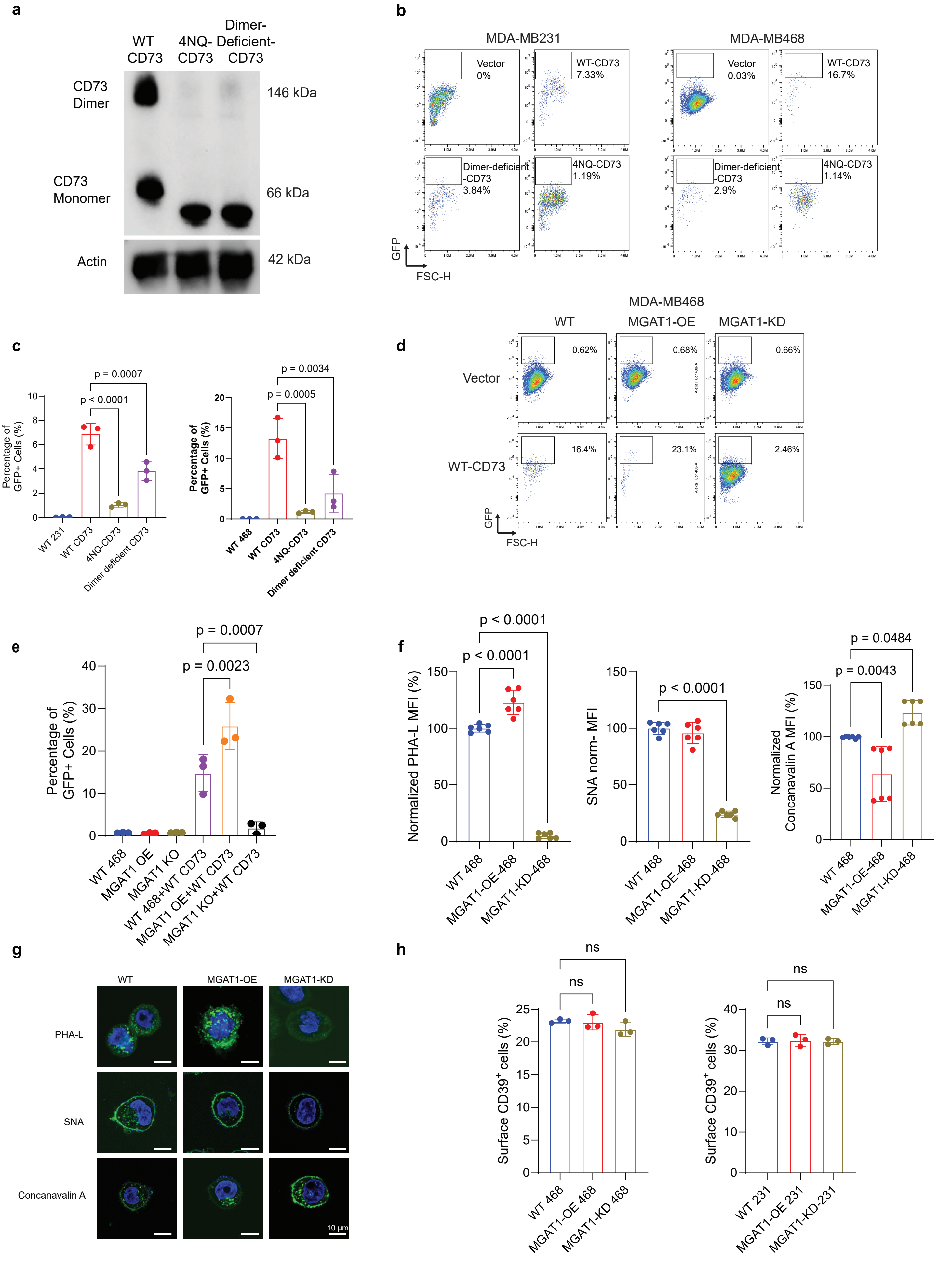
a
WT CD73
4NQ- CD73
Dimer- Deficient- CD73
CD73 Dimer
CD73 Monomer
Actin
146 kDa
66 kDa
42 kDa
b
MDA-MB231
MDA-MB468
Vector 0%
WT-CD73 7.33%
Dimer-deficient -CD73 3.84%
4NQ-CD73 1.19%
Vector 0.03%
WT-CD73 16.7%
Dimer-deficient -CD73 2.9%
4NQ-CD73 1.14%
GFP
FSC-H
c
p = 0.0007
p < 0.0001
Percentage of GFP+ Cells (%)
WT 231
WT CD73
4NQ-CD73
Dimer deficient CD73
p = 0.0034
p = 0.0005
WT 468
d
MDA-MB468
WT
MGAT1-OE
MGAT1-KD
Vector
WT-CD73
0.62%
0.68%
0.66%
16.4%
23.1%
2.46%
e
p = 0.0007
p = 0.0023
Percentage of GFP+ Cells (%)
WT 468
MGAT1 OE
MGAT1 KO
WT 468+WT CD73
MGAT1 OE+WT CD73
MGAT1 KO+WT CD73
f
p < 0.0001
p < 0.0001
Normalized PHA-L MFI (%)
WT 468
MGAT1-OE-468
MGAT1-KD-468
p < 0.0001
SNA norm- MFI
p = 0.0484
p = 0.0043
Normalized Concanavalin A MFI (%)
g
WT
MGAT1-OE
MGAT1-KD
PHA-L
SNA
Concanavalin A
10 μm
h
ns
ns
Surface CD39+ cells (%)
WT 468
MGAT1-OE 468
MGAT1-KD 468
WT 231
MGAT1-OE 231
MGAT1-KD-231

Supplementary Fig. 7: Investigate MGAT1-orchestrated CD73 dimerization with Split-GFP system. **a** The dimerization of WT CD73, CD73-4NQ, or CD73Δ480-537 was determined by immunoblotting of semi-native gels. **b, c** The Split-GFP signal generated from dimerized WT CD73, $CD73^{N53Q/N311Q/N333Q/N403Q,}$ or 480-537 truncated CD73 was measured by flow cytometry analysis in MDA-MB468 and MDA-MB231 cells (**b**) and the quantification is listed (**c**). **d, e** The Split-GFP signal generated from dimerized WT-CD73 was measured by flow cytometry analysis in MGAT1-WT/OE/KD MDA-MB-468 (**d**) and the quantification is listed (**e**). **f** The normalized mean fluorescence intensity (MFI) of lectin staining (PLA-H, SNA, and concanavalin A) on MGAT1-WT/OE/KD-MDA-MB468 were measured and quantified using flow cytometry. ***$P < 0.0001$, ****$P < 0.0001$. Data (means ± SEM) are representative of at least three independent experiments. **g** The complex type N-glycans, sialic acids, and high mannose type of glycans were detected by lectin immunofluorescence staining (PLA-H, SNA, and concanavalin A) on MGAT1-WT/OE/KD-MDA-MB468 with confocal imaging. **h** Membrane CD39 was measured with flow cytometry in MDA-MB468 and MDA-MB231 cells with MGAT1 OE/KD. Data (represented as means ± SEM), western blot and flow cytometry are representative of three independent experiments. Statistical significance was determined using one-way ANOVA with Tukey's multiple comparisons test (**c**, **e**, **f**, **h**). Source data are provided as a Source Data file.

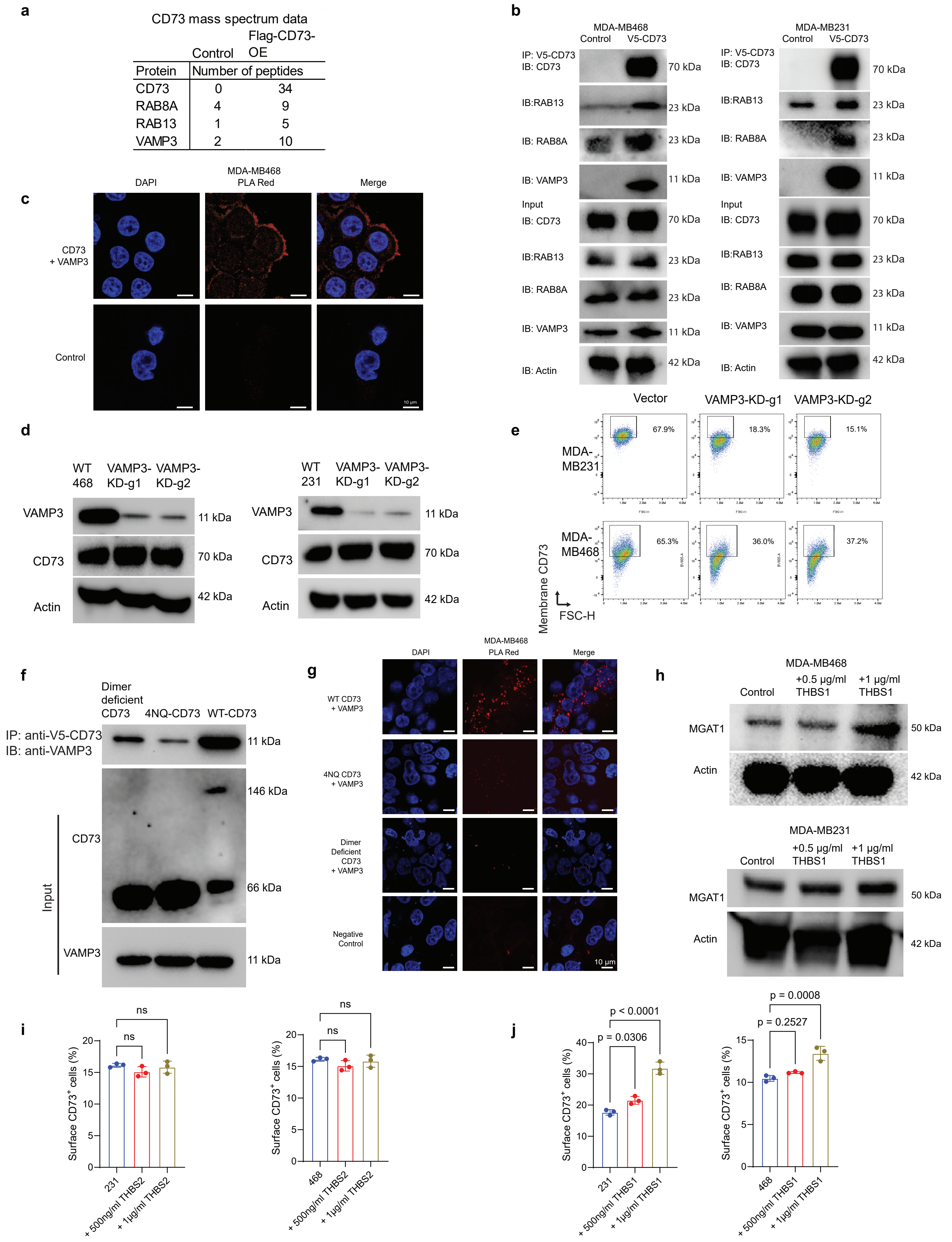
a
CD73 mass spectrum data
Control
Flag-CD73-OE
Protein
Number of peptides
CD73 0 34
RAB8A 4 9
RAB13 1 5
VAMP3 2 10
b
MDA-MB468
MDA-MB231
Control
V5-CD73
IP: V5-CD73
IB: CD73
IB:RAB13
IB: RAB8A
IB: VAMP3
Input
IB: CD73
IB: Actin
70 kDa
23 kDa
11 kDa
42 kDa
c
DAPI
MDA-MB468
PLA Red
Merge
CD73
+ VAMP3
Control
10 μm
d
WT 468
VAMP3-KD-g1
VAMP3-KD-g2
WT 231
VAMP3
CD73
Actin
11 kDa
70 kDa
42 kDa
e
Vector
VAMP3-KD-g1
VAMP3-KD-g2
MDA-MB231
67.9%
18.3%
15.1%
MDA-MB468
65.3%
36.0%
37.2%
Membrane CD73
FSC-H
f
Dimer deficient CD73
4NQ-CD73
WT-CD73
IP: anti-V5-CD73
IB: anti-VAMP3
11 kDa
146 kDa
66 kDa
Input
CD73
VAMP3
g
DAPI
MDA-MB468
PLA Red
Merge
WT CD73
+ VAMP3
4NQ CD73
+ VAMP3
Dimer
Deficient
CD73
+ VAMP3
Negative
Control
10 μm
h
MDA-MB468
Control
+0.5 μg/ml THBS1
+1 μg/ml THBS1
MGAT1
Actin
50 kDa
42 kDa
MDA-MB231
i
Surface CD73+ cells (%)
ns
231
+ 500ng/ml THBS2
+ 1μg/ml THBS2
468
j
p < 0.0001
p = 0.0306
+ 500ng/ml THBS1
+ 1μg/ml THBS1
p = 0.0008
p = 0.2527

Supplementary Fig. 8: THBS1-MGAT1 axis was discovered in regulating CD73 translocation to membrane through VAMP3. a The List of transport proteins identified by MS analysis. **b** The biochemical interaction between CD73 and potential transport proteins was validated by coimmunoprecipitation of V5-CD73 in MDA-MB468 and MDA-MB231 cells. The samples derived from the same experiment but different gels for CD73, RAB13, RAB8A, and VAMP3, and β-ACTIN were processed in parallel. **c** The intracellular interaction between CD73 and VAMP3 was validated with Proximity Ligation Assay (PLA-red) with anti-VAMP3 and anti-CD73 antibodies or control IgG followed by confocal imaging. **d** The protein level of VAMP3 and CD73 was determined by immunoblotting in WT MDA-MB468 and MDA-MB468-VAMP3-KD breast cancer stable cells. **e** The percentage of membrane $CD73^+$ cells was measured with flow cytometry in WT MDA-MB231 and MDA-MB468-VAMP3-KD. ****$p < 0.0001$. Data (mean ± SEM) are representative of at least three independent experiments. **f** The biochemical interaction between VAMP3 and CD73 monomer or CD73 dimer was investigated by coimmunoprecipitation of V5-CD73, V5-$CD73^{N53Q/N311Q/N333Q/N403Q}$ and dimer-deficient CD73 in HEK-293T cells. **g** The intracellular interaction between WT CD73/$CD73^{N53Q/N311Q/N333Q/N403Q}$/dimer-deficient CD73 and VAMP3 was validated with Proximity Ligation Assay (PLA-red) with anti-VAMP3 and anti-CD73 antibodies or control IgG followed by confocal imaging. **h** The protein levels of MGAT1 in MDA-MB468 and MDA-MB231 after treatment of THBS1 at indicated concentrations were determined by immunoblotting. **i, j** The fraction of membrane $CD73^+$ cells was measured with flow cytometry in MDA-MB468 and MDA-MB231 after treatment of THBS2 (**i**) or THBS1 (**j**) at indicated concentrations. Data (represented as means ± SEM), images, western blot and flow cytometry are representative of three independent experiments. Statistical significance was determined using one-way ANOVA with Tukey's multiple comparisons test (**i**, **j**). Source data are provided as a Source Data file.

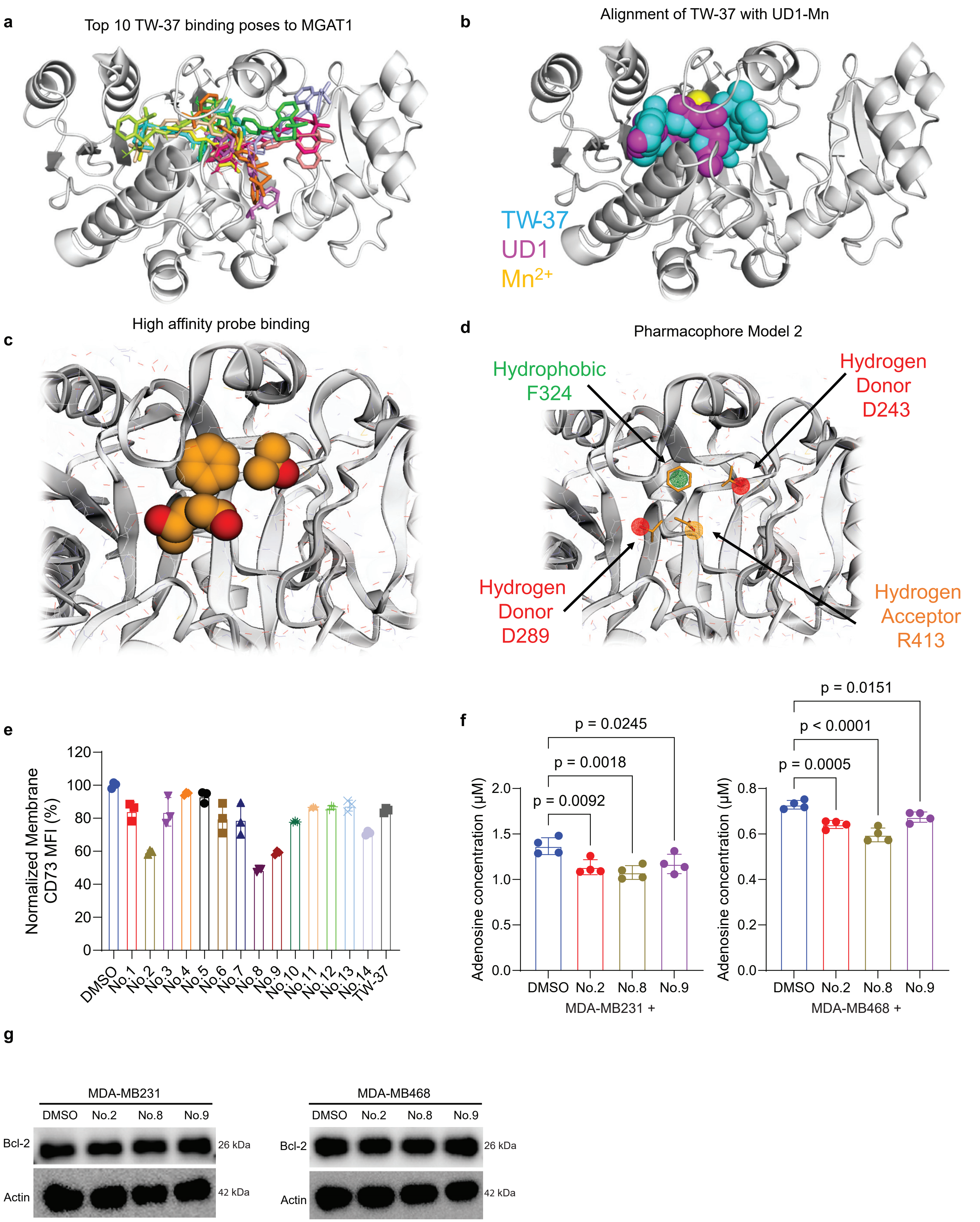

a
Top 10 TW-37 binding poses to MGAT1
b
Alignment of TW-37 with UD1-Mn
TW-37
UD1
Mn2+
c
High affinity probe binding
d
Pharmacophore Model 2
Hydrophobic F324
Hydrogen Donor D243
Hydrogen Donor D289
Hydrogen Acceptor R413
e
Normalized Membrane CD73 MFI (%)
120
100
80
60
40
20
0
DMSO
No.1
No.2
No.3
No.4
No.5
No.6
No.7
No.8
No.9
No.10
No.11
No.12
No.13
No.14
TW-37
f
Adenosine concentration (μM)
2.0
1.5
1.0
0.5
0.0
p = 0.0245
p = 0.0018
p = 0.0092
DMSO
No.2
No.8
No.9
MDA-MB231 +
Adenosine concentration (μM)
0.8
0.6
0.4
0.2
0.0
p = 0.0151
p < 0.0001
p = 0.0005
DMSO
No.2
No.8
No.9
MDA-MB468 +
g
MDA-MB231
DMSO
No.2
No.8
No.9
Bcl-2
26 kDa
Actin
42 kDa
MDA-MB468
DMSO
No.2
No.8
No.9
Bcl-2
26 kDa
Actin
42 kDa

Supplementary Fig. 9: Combination of molecular docking, druggability simulations, and pharmacophore modeling, for identification of MGAT1-targeting compounds. **a** Top 10 binding poses of TW-37 to MGAT1, predicted by AutoDock Vina. Different binding pose of TW-37 is colored differently. **b** Comparison of the most energy favorable binding pose of TW-37 (*cyan* spheres) with the resolved Uridine-diphosphate-N-acetylglucosamine (UD1; *purple* spheres) and Mn^{2+} (*yellow* sphere). The most favorable binding affinity of TW-37 was computed to be -9.4 kcal/mol by Vina. The binding pose of UD1 and Mn^{2+} was adopted from the resolved MGAT1 homolog (PDB: 1foa). **c** High affinity probe binding pose identified by Pharmmaker from druggability simulations. **d** Construction of pharmacophore model 2 (PM_2) based on high affinity probe binding and high affinity binding residues identified by Pharmmaker. **e** The MFI of membrane CD73 in MDA-MB231 under treatment of 15 screened compounds was measured with flow cytometry. **f** MDA-MB231 and MDA-MB468 cells were treated with indicated compounds and adenosine productions were determined by adenosine assay kit. **g** The protein levels of Bcl-2 in MDA-MB468 and MDA-MB231 after treatment of DMSO, compound No.2, No.8 and No.9 at were determined by immunoblotting. Data (represented as means ± SEM) and western blots are representative of three independent experiments. Statistical significance was determined using one-way ANOVA with Tukey's multiple comparisons test**.** Source data are provided as a Source Data file.

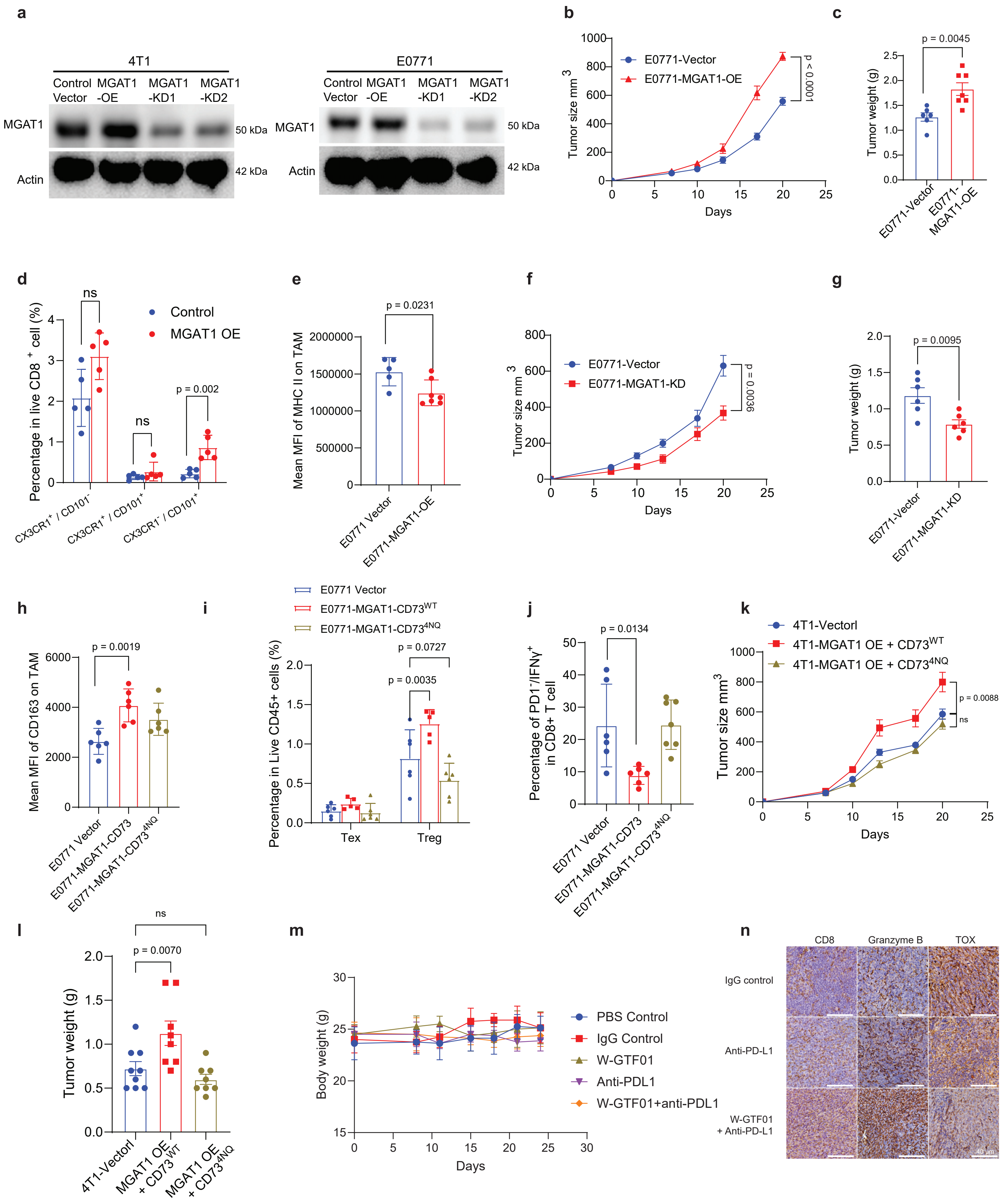

a
4T1
Control Vector
MGAT1 -OE
MGAT1 -KD1
MGAT1 -KD2
MGAT1
Actin
50 kDa
42 kDa
E0771
b
E0771-Vector
E0771-MGAT1-OE
p < 0.0001
Tumor size mm3
Days
c
p = 0.0045
Tumor weight (g)
d
ns
p = 0.002
Control
MGAT1 OE
Percentage in live CD8+ cell (%)
CX3CR1+ / CD101-
CX3CR1+ / CD101+
CX3CR1- / CD101+
e
p = 0.0231
Mean MFI of MHC II on TAM
E0771 Vector
E0771-MGAT1-OE
f
E0771-MGAT1-KD
p = 0.0036
g
p = 0.0095
h
p = 0.0019
Mean MFI of CD163 on TAM
E0771-MGAT1-CD73
E0771-MGAT1-CD73 4NQ
i
E0771-MGAT1-CD73WT
E0771-MGAT1-CD734NQ
p = 0.0727
p = 0.0035
Percentage in Live CD45+ cells (%)
Tex
Treg
j
p = 0.0134
Percentage of PD1-/IFNγ+ in CD8+ T cell
k
4T1-Vectorl
4T1-MGAT1 OE + CD73WT
4T1-MGAT1 OE + CD734NQ
p = 0.0088
ns
l
p = 0.0070
MGAT1 OE + CD73WT
MGAT1 OE + CD734NQ
m
Body weight (g)
PBS Control
IgG Control
W-GTF01
Anti-PDL1
W-GTF01+anti-PDL1
n
CD8
Granzyme B
TOX
IgG control
Anti-PD-L1
W-GTF01 + Anti-PD-L1

Supplementary Fig. 10: MGAT1-mediated glycosylation of CD73 orchestrates tumor immune evasion in vivo. a MGAT1 protein levels in 4T1 and E0771 cells with MGAT1 OE and MGAT1 KD were determined by immunoblotting. **b, c** Tumor growth (**b**) and endpoint tumor weight (**c**) in E0771-MGAT1 OE vs. control tumors in C57BL/6 WT mice. **d** Percentages of $CX3CR1^+/CD101^-$, $CX3CR1^+/CD101^+$, and $CX3CR1^-/CD101^+$ $CD8^+$ T cells in 4T1-MGAT1 OE vs. control tumors. **e** Membrane MHC II expression in TAMs from E0771-MGAT1 OE vs. control tumors. **f, g** Tumor growth (**f**) and endpoint tumor weight (**g**) in E0771-MGAT1 KD vs. control tumors. **h** CD163 expression in TAMs from E0771-MGAT1 OE-CD73 WT, E0771-MGAT1 OE-$CD73^{N53Q/N311Q/N333Q/N403Q}$, and control tumors. **i** Percentages of infiltrated immune populations ($CD45^+$ cells) in E0771-MGAT1 OE + $CD73^{WT}$, E0771-MGAT1 OE + $CD73^{N53Q/N311Q/N333Q/N403Q}$, and control tumors. **j** IFNγ+/PD1- CD8+ T cell percentages in tumor infiltrates of E0771-MGAT1 OE + $CD73^{WT}$, E0771-MGAT1 OE + $CD73^{N53Q/N311Q/N333Q/N403Q}$, and control tumors. **k, l** Tumor growth (k) and endpoint tumor weight (l) in 4T1-MGAT1-$CD73^{WT}$, 4T1-MGAT1-$CD73^{N53Q/N311Q/N333Q/N403Q}$, and control tumors in BALB/c WT mice. **m** Body weight of 4T1-hPD-L1 tumor-bearing mice treated with W-GTF01 (10 mg/kg, i.p.) twice/week and durvalumab (10 mg/kg, i.p.) three times/week, with PBS/IgG as controls. **n** Representative IHC images of CD8, Granzyme B, and TOX in mouse tumors treated with IgG, anti-PD-L1, or W-GTF01 + anti-PD-L1. Scale bar = 40 μm. Data (means ± SEM), western blot and flow cytometry are representative of at least three independent experiments with 5-10 independently analyzed mice per group. Statistical significance was determined using one-way ANOVA with Tukey's multiple comparisons test (**h-j**, **l**) or two-tailed unpaired t test (**c-e**, **g**). For tumor growth statistical analysis (**b**, **f**, **k**), two-way ANOVAs followed by Tukey's multiple comparison tests were performed. Source data are provided as a Source Data file.

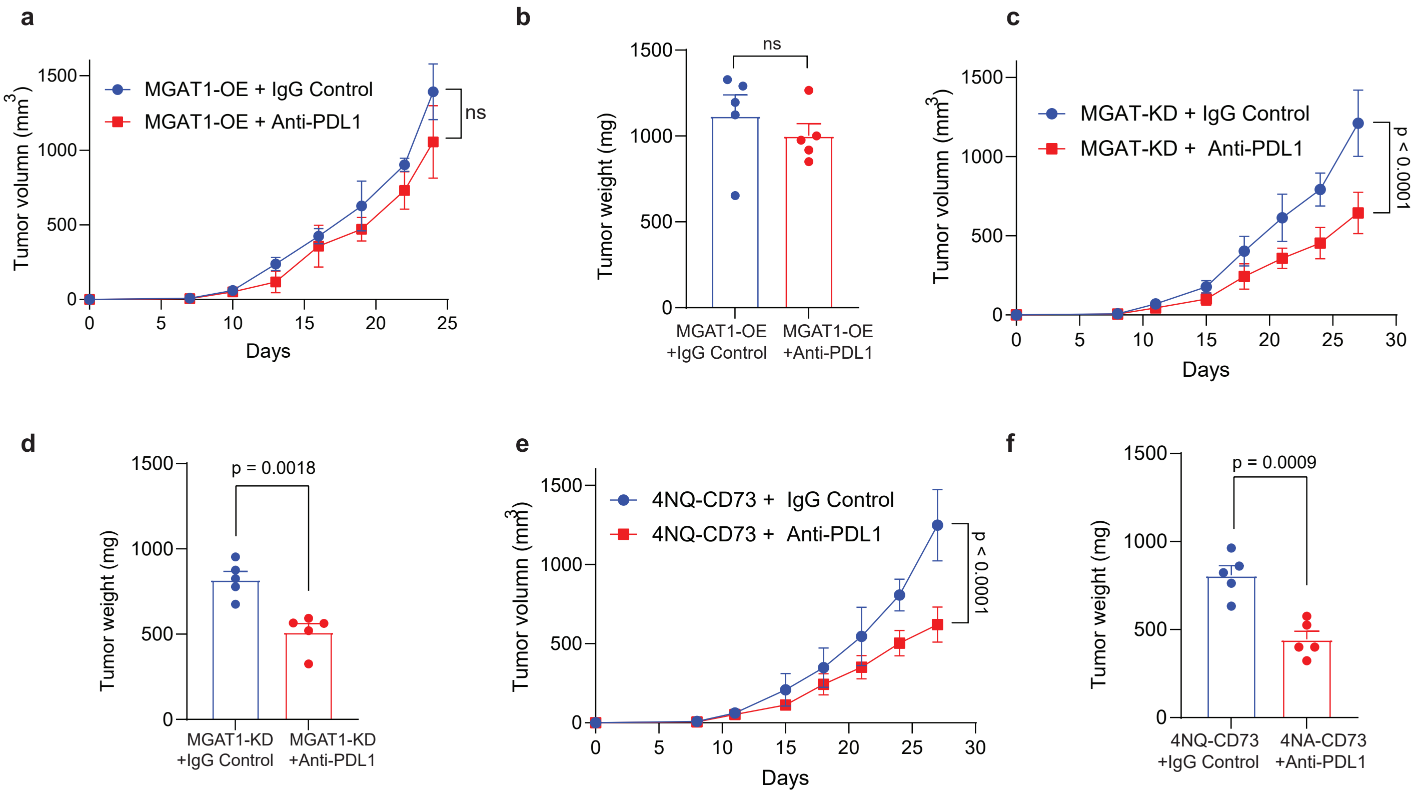

Supplementary Fig. 11: The gain- and loss-of-function experiments with MGAT1-KD/OE and CD73-4NQ to validate the critical role of MGAT1-mediated CD73 glycosylation in the resistance to ICB therapy. a-f 4T1-hPD-L1-MGAT1-OE, 4T1-hPD-L1-MGAT1-KD, 4T1-hPD-L1-4NQ-CD73 cells, where the endogenous mouse PD-L1 was replaced with its human counterpart, were orthotopically injected into the left fourth mammary fat pad and allowed to grow to ~100 mm^3, followed by injection of PD-L1 antibody durvalumab (10 mg/kg, i.p.) 3 times/week. PBS and IgG were used in the control groups. The tumor growth (**a**, **c**, **e**) of the mice were plotted and the tumor weight were measured (**b, d, f**). Data (means ± SEM) are representative of at least three independent experiments with 5-10 independently analyzed mice per group. Statistical significance was determined using two-tailed unpaired t test (**b**, **d**, **f**). For tumor growth statistical analysis (**a**, **c**, **e**), two-way ANOVAs followed by Tukey's multiple comparison tests were performed. Source data are provided as a Source Data file.

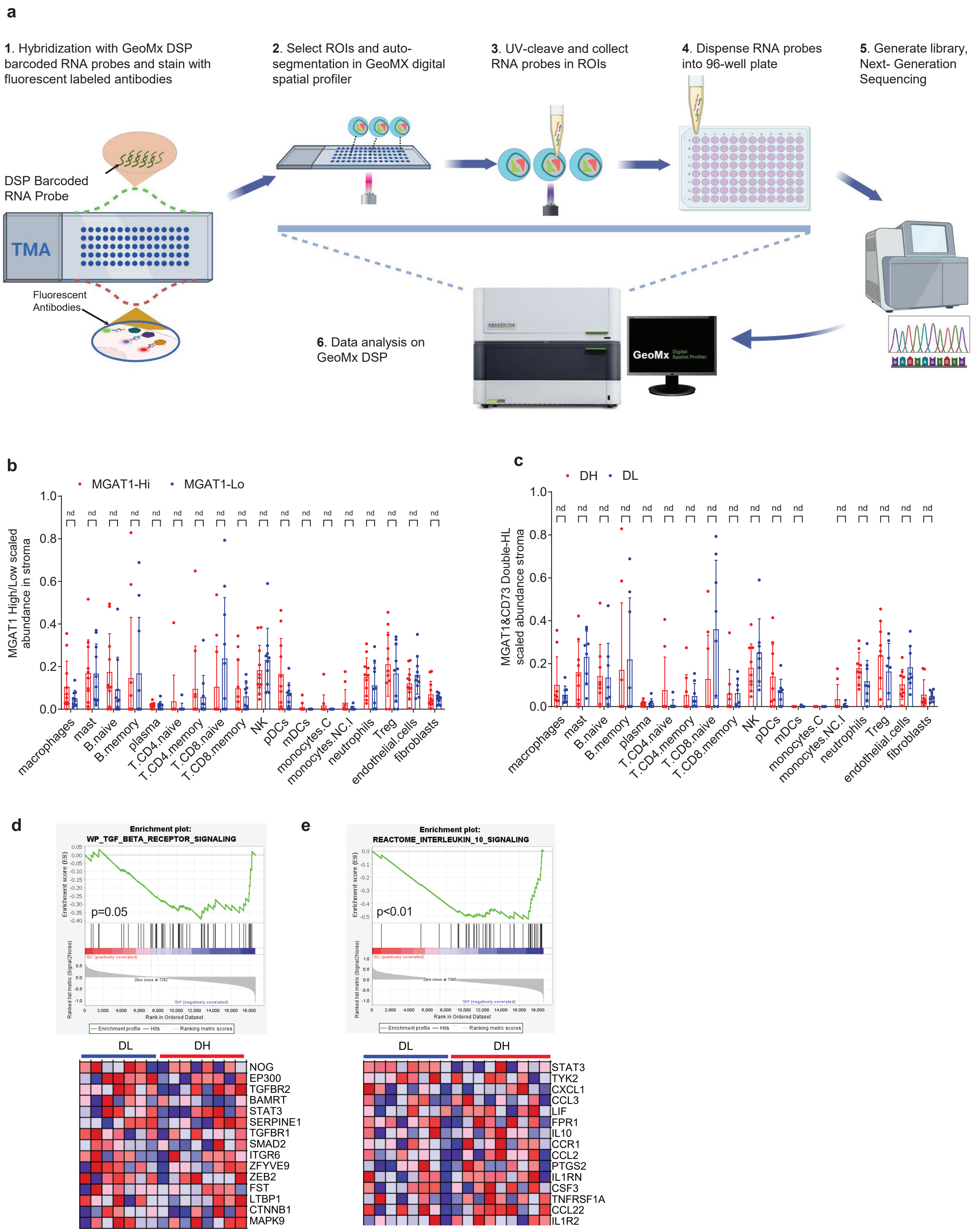
a
1. Hybridization with GeoMx DSP barcoded RNA probes and stain with fluorescent labeled antibodies
2. Select ROIs and auto-segmentation in GeoMX digital spatial profiler
3. UV-cleave and collect RNA probes in ROIs
4. Dispense RNA probes into 96-well plate
5. Generate library, Next- Generation Sequencing
DSP Barcoded RNA Probe
TMA
Fluorescent Antibodies
6. Data analysis on GeoMx DSP
GeoMx
b
MGAT1-Hi
MGAT1-Lo
MGAT1 High/Low scaled abundance in stroma
nd
macrophages
mast
B.naive
B.memory
plasma
T.CD4.naive
T.CD4.memory
T.CD8.naive
T.CD8.memory
NK
pDCs
mDCs
monocytes.C
monocytes.NC.I
neutrophils
Treg
endothelial.cells
fibroblasts
c
DH
DL
MGAT1&CD73 Double-HL scaled abundance stroma
d
Enrichment plot: WP_TGF_BETA_RECEPTOR_SIGNALING
p=0.05
Enrichment score (ES)
Rank in Ordered Dataset
Enrichment profile
Hits
Ranking metric scores
e
Enrichment plot: REACTOME_INTERLEUKIN_10_SIGNALING
p<0.01
DL
DH
NOG
EP300
TGFBR2
BAMRT
STAT3
SERPINE1
TGFBR1
SMAD2
ITGR6
ZFYVE9
ZEB2
FST
LTBP1
CTNNB1
MAPK9
STAT3
TYK2
CXCL1
CCL3
LIF
FPR1
IL10
CCR1
CCL2
PTGS2
IL1RN
CSF3
TNFRSF1A
CCL22
IL1R2

Supplementary Fig. 12: The signature of MGAT-CD73 double-high is associated with unfavorable immune response. a The experimental flow diagram of spatial profiling using GeoMx® Digital Spatial Profiler (DSP). Created in BioRender. Zhang, B. (2025) https://BioRender.com/z86g900. **b, c** CIBERSORT analysis of the relative abundance of individual cell populations between MGAT1lo and MGAT1hi areas (**b**) or between MGAT1loCD73lo (DL) and MGAT1hiCD73hi areas (DH) (**c**) among PanCK^{+} tumor compartments using GeoMx-DSP transcriptomics data. **d, e** Gene set enrichment analysis (GSEA) demonstrating positive enrichment of hallmark curated gene sets for TGF-β signaling (**d**) in non-tumor areas and IL-10 signaling (**e**) in tumor areas of DH cases compared to DL cases. Data (means ± SEM) are representative of at least three independent experiments. Statistical significance was determined using two-tailed unpaired t test (**b**, **c**). Source data are provided as a Source Data file.

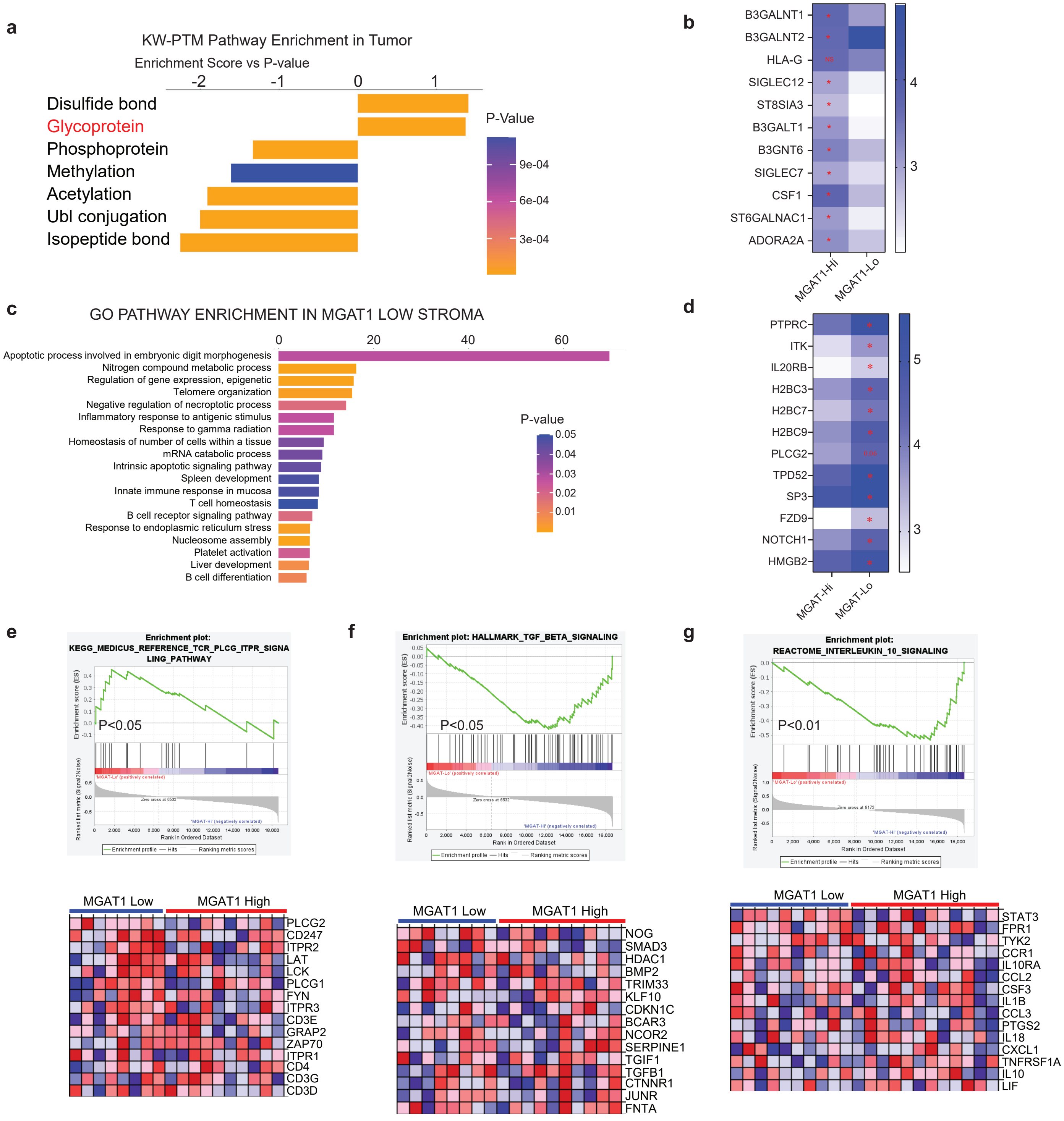
a
KW-PTM Pathway Enrichment in Tumor
Enrichment Score vs P-value
-2
-1
0
1
Disulfide bond
Glycoprotein
Phosphoprotein
Methylation
Acetylation
Ubl conjugation
Isopeptide bond
P-Value
9e-04
6e-04
3e-04
b
B3GALNT1
B3GALNT2
HLA-G
SIGLEC12
ST8SIA3
B3GALT1
B3GNT6
SIGLEC7
CSF1
ST6GALNAC1
ADORA2A
4
3
MGAT1-Hi
MGAT1-Lo
c
GO PATHWAY ENRICHMENT IN MGAT1 LOW STROMA
0
20
40
60
Apoptotic process involved in embryonic digit morphogenesis
Nitrogen compound metabolic process
Regulation of gene expression, epigenetic
Telomere organization
Negative regulation of necroptotic process
Inflammatory response to antigenic stimulus
Response to gamma radiation
Homeostasis of number of cells within a tissue
mRNA catabolic process
Intrinsic apoptotic signaling pathway
Spleen development
Innate immune response in mucosa
T cell homeostasis
B cell receptor signaling pathway
Response to endoplasmic reticulum stress
Nucleosome assembly
Platelet activation
Liver development
B cell differentiation
P-value
0.05
0.04
0.03
0.02
0.01
d
PTPRC
ITK
IL20RB
H2BC3
H2BC7
H2BC9
PLCG2
TPD52
SP3
FZD9
NOTCH1
HMGB2
5
4
3
MGAT-Hi
MGAT-Lo
e
Enrichment plot:
KEGG_MEDICUS_REFERENCE_TCR_PLCG_ITPR_SIGNALING_PATHWAY
P<0.05
f
Enrichment plot: HALLMARK_TGF_BETA_SIGNALING
P<0.05
g
Enrichment plot:
REACTOME_INTERLEUKIN_10_SIGNALING
P<0.01
MGAT1 Low
MGAT1 High
PLCG2
CD247
ITPR2
LAT
LCK
PLCG1
FYN
ITPR3
CD3E
GRAP2
ZAP70
ITPR1
CD4
CD3G
CD3D
MGAT1 Low
MGAT1 High
NOG
SMAD3
HDAC1
BMP2
TRIM33
KLF10
CDKN1C
BCAR3
NCOR2
SERPINE1
TGIF1
TGFB1
CTNNB1
JUNR
FNTA
MGAT1 Low
MGAT1 High
STAT3
FPR1
TYK2
CCR1
IL10RA
CCL2
CSF3
IL1B
CCL3
PTGS2
IL18
CXCL1
TNFRSF1A
IL10
LIF

Supplementary Fig. 13: The signature of MGAT1lo is associated with favorable immune response.

a, b UP-KW functional annotation (UniProt KeyWord Functional Annotations) analysis of PTM on the DEGs in PanCK$^+$ tumor epithelial compartment between MGAT1lo cases and MGAT1hi cases (A), revealing selected Glycoprotein at increased levels in MGAT1hi tumors (B). **c, d** GO Pathway analysis of DEGs in non-tumor areas between MGAT1lo cases and MGAT1hi cases, highlighting selected genes involving Innate immune responses and B cell receptor signaling at increased levels in MGAT1lo cases. **e-g** Gene set enrichment analysis (GSEA) demonstrating positive enrichment of TCR signaling pathway (**e**) accompanied by decreased enrichment for TGF-β signaling (**f**) in non-tumor areas and IL-10 signaling (**g**) in tumor areas of MGAT1lo cases compared to MGAT1hi cases. Source data are provided as a Source Data file.

a

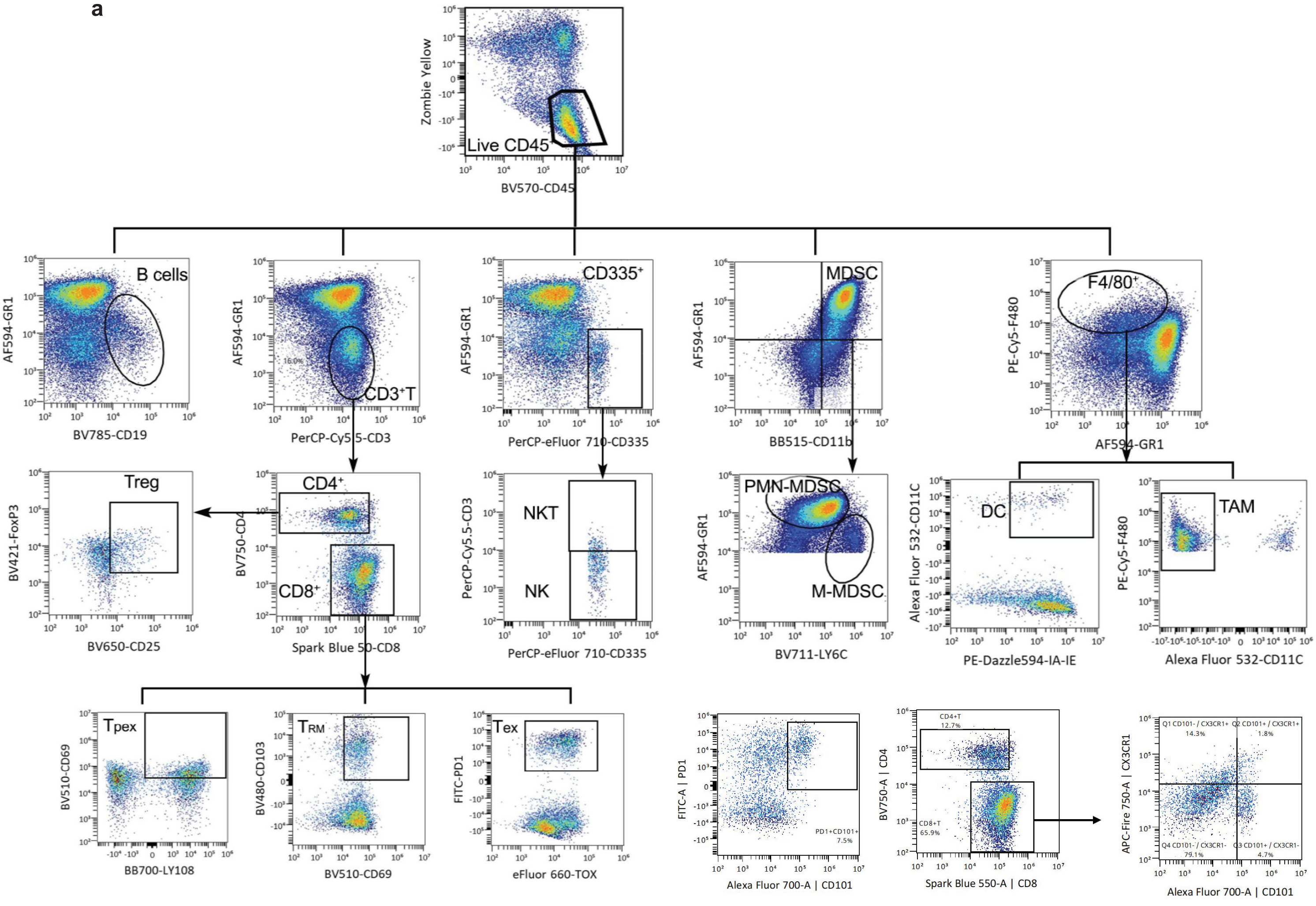

Supplementary Fig. 14. Gating strategy. a Gating strategy for the Cytek spectral flow cytometry data analysis in tumor infiltrated lymphocytes (Fig. 7e).
