# Supplementary material for: MGAT1-Guided complex N-Glycans on CD73 regulate immune evasion in triple-negative breast cancer
Source: Nat Commun. 2025 Apr 15;16:3552. doi: 10.1038/s41467-025-58524-9 (PMC11997035; doi:10.1038/s41467-025-58524-9)
Supplement: Supplementary file 2 — Description of Additional Supplementary Information [file 41467_2025_58524_MOESM2_ESM.docx]

**Description of Additional Supplementary Files**

**Supplementary Data Legends:**

**Supplementary Data 1:** List of potential binding partners of MGAT1 identified from MGAT1 mass spectrometry analysis.

**Supplementary Data 2**: A list of compounds tested in this study

**Supplementary Data 3:** Antibodies used in this study
