# Supplementary material for: MGAT1-Guided complex N-Glycans on CD73 regulate immune evasion in triple-negative breast cancer
Source: Nat Commun. 2025 Apr 15;16:3552. doi: 10.1038/s41467-025-58524-9 (PMC11997035; doi:10.1038/s41467-025-58524-9)
Supplement: Supplementary file 6 — Reporting Summary [file 41467_2025_58524_MOESM6_ESM.pdf]

nature portfolio

Corresponding author(s): Ivet Bahar, Bin Zhang, Yong Wan

Last updated by author(s): Mar 11, 2025

Reporting Summary

Nature Portfolio wishes to improve the reproducibility of the work that we publish. This form provides structure for consistency and transparency in reporting. For further information on Nature Portfolio policies, see our Editorial Policies and the Editorial Policy Checklist.

Statistics

For all statistical analyses, confirm that the following items are present in the figure legend, table legend, main text, or Methods section.

n/a	Confirmed	
☐	☒	The exact sample size (*n*) for each experimental group/condition, given as a discrete number and unit of measurement
☐	☒	A statement on whether measurements were taken from distinct samples or whether the same sample was measured repeatedly
☐	☒	The statistical test(s) used AND whether they are one- or two-sided<br>*Only common tests should be described solely by name; describe more complex techniques in the Methods section.*
☐	☒	A description of all covariates tested
☒	☐	A description of any assumptions or corrections, such as tests of normality and adjustment for multiple comparisons
☐	☒	A full description of the statistical parameters including central tendency (e.g. means) or other basic estimates (e.g. regression coefficient) AND variation (e.g. standard deviation) or associated estimates of uncertainty (e.g. confidence intervals)
☐	☒	For null hypothesis testing, the test statistic (e.g. *F*, *t*, *r*) with confidence intervals, effect sizes, degrees of freedom and *P* value noted<br>*Give P values as exact values whenever suitable.*
☒	☐	For Bayesian analysis, information on the choice of priors and Markov chain Monte Carlo settings
☒	☐	For hierarchical and complex designs, identification of the appropriate level for tests and full reporting of outcomes
☐	☒	Estimates of effect sizes (e.g. Cohen's *d*, Pearson's *r*), indicating how they were calculated

Our web collection on statistics for biologists contains articles on many of the points above.

Software and code

Policy information about availability of computer code

Data collection

Flow cytometry data was collected using FACSDiva software, all IF images were captured and processed by Leica software and Lionheart software. ProDy for trajectory analysis, publicly accessible and deposited in GitHub repository （https://github.com/prody/ProDy）
DruGUI for druggability simulations, publicly available in our ProDy Application Programming Interface (API)
Pharmmaker, for pharmacophore modeling, publicly available in our ProDy API
NAMD for molecular dynamics (MD) simulations, publicly available
ClusPro and AutoDock Vina for docking simulations, publicly available
Pharmit for virtual screening, publicly available

Data analysis

All statistical analysis were done using Graphpad prism 10.0. The flow cytometry data were processed using Flowjo V10. All IHC or IF images were processed by Fuji, ImageJ, and Imaris
ProDy for trajectory analysis, publicly accessible and deposited in GitHub repository （https://github.com/prody/ProDy）
DruGUI for druggability simulations, publicly available in our ProDy Application Programming Interface (API)
Pharmmaker, for pharmacophore modeling, publicly available in our ProDy API
NAMD for molecular dynamics (MD) simulations, publicly available
ClusPro and AutoDock Vina for docking simulations, publicly available
Pharmit for virtual screening, publicly available

For manuscripts utilizing custom algorithms or software that are central to the research but not yet described in published literature, software must be made available to editors and reviewers. We strongly encourage code deposition in a community repository (e.g. GitHub). See the Nature Portfolio guidelines for submitting code & software for further information.

Data

Policy information about availability of data

All manuscripts must include a data availability statement. This statement should provide the following information, where applicable:

- Accession codes, unique identifiers, or web links for publicly available datasets
- A description of any restrictions on data availability
- For clinical datasets or third party data, please ensure that the statement adheres to our policy

All data supporting the findings of this study are available within the paper and its Supplementary Information.

Data availability

The authors declare that all data supporting the findings of this study are available within the article and its supplementary information. The mass spectrometry proteomics data generated in this study have been deposited to the ProteomeXchange Consortium via the PRIDE partner repository with the dataset identifier PXD055157. The proteomic data utilized in this research are publicly accessible through Breast cancer proteomic data through National Cancer Institute Clinical Proteomic Tumor Analysis Consortium (https://proteomic.datacommons.cancer.gov/pdc/; accession number: PDC000173). TNBC immune-related mRNA data utilized in this research can be accessed through Gene Expression Omnibus database (accession number: GSE88847). Immune analysis using CAMOIP, TIMER2 and TIDE online platform are available with following links:
https://www.camoip.net, http://timer.cistrome.org and http://tide.dfci.harvard.edu/login/. Source data are provided with this paper.

The mass spectrometry proteomics data have been deposited to the ProteomeXchange Consortium via the PRIDE partner repository with the dataset identifier PXD055157.
Submission details:

Project Name: MGAT1-Mediated Glycosylation Orchestrates Immune Checkpoints and Antitumor Immunity

Project accession: PXD055157

Project DOI: Not applicable

Reviewer account details:

Reviewer login website: https://www.ebi.ac.uk/pride/login

Reviewer Username: reviewer_pxd055157@ebi.ac.uk

Password: zqQmJ6yhmU86

Research involving human participants, their data, or biological material

Policy information about studies with human participants or human data. See also policy information about sex, gender (identity/presentation), and sexual orientation and race, ethnicity and racism.

Reporting on sex and gender	No human research in this manuscript.
Reporting on race, ethnicity, or other socially relevant groupings	No human research in this manuscript.
Population characteristics	No human research in this manuscript.
Recruitment	No human research in this manuscript.
Ethics oversight	No human research in this manuscript.

Note that full information on the approval of the study protocol must also be provided in the manuscript.

Field-specific reporting

Please select the one below that is the best fit for your research. If you are not sure, read the appropriate sections before making your selection.

☒ Life sciences ☐ Behavioural & social sciences ☐ Ecological, evolutionary & environmental sciences

For a reference copy of the document with all sections, see nature.com/documents/nr-reporting-summary-flat.pdf

Life sciences study design

All studies must disclose on these points even when the disclosure is negative.

Sample size	For the publicly available datasets, all available samples were included. All in vitro experiments were performed with at least n=6, comprising 3 independent biological replicates. All in vivo experiments were conducted with a minimum sample size of 5 mice per condition.
Data exclusions	There was no data exclusion in the analysis performed in this work.
Replication	All experiments that are included in this work were replicated at least 3 times.
Randomization	For in vivo experiments, the mice were randomly assigned to receive either treatment or tumor implantation. After receiving the treatment or injection, they were placed in separate cages according to their experimental group.
Blinding	For in vivo experiments, once the tumors were removed, subsequent analyses were performed in a blinded manner.

Reporting for specific materials, systems and methods

We require information from authors about some types of materials, experimental systems and methods used in many studies. Here, indicate whether each material, system or method listed is relevant to your study. If you are not sure if a list item applies to your research, read the appropriate section before selecting a response.

Materials & experimental systems

n/a	Involved in the study	
☐	☒	Antibodies
☐	☒	Eukaryotic cell lines
☒	☐	Palaeontology and archaeology
☐	☒	Animals and other organisms
☒	☐	Clinical data
☒	☐	Dual use research of concern
☒	☐	Plants

Methods

n/a	Involved in the study	
☒	☐	ChIP-seq
☐	☒	Flow cytometry
☒	☐	MRI-based neuroimaging

Antibodies

Antibodies used

MGAT1 polyclonal antibody Sigma-Aldrich SAB1400165)
MGAT1 polyclonal antibody Proteintech, 15103-AP
NT5E/CD73 (D7F9A) Rabbit mAB Cell Signaling Technology #13160
CD73 monoclonal antibody (4G4) NBP1-60135
Monoclonal Anti-Flag M2 antibody produced in mouse Sigma F3165
Anti-V5 polyclonal antibody produced in rabbit Sigma V8137
Anti-GM-130 (C-terminal) Polyclonal antibody Sigma G7295
VAMP3 Polyclonal antibody Invitrogen PA1-767A
RAB8A Polyclonal antibody Invitrogen PA5-72625
RAB13 Polyclonal antibody biotechne MAB8305
Na,K-ATPase Antibody Cell Signaling #3010
β-Actin (13E5) Rabbit mAb (HRP Conjugate) Cell Signaling #5125S
CD4-BV750 (GK1.5) Biolegend 100467
CD8a-Spark Blue550 (53-6.7) Biolegend 100780
CD45-BV570 (30-F11) Biolegend 103135
CD3e-Percp5.5 (145-2C11) Biolegend 100328
CD11b-BB515 (M1/70) BD Horizon 564454
CD11c-AF532 (N418) eBiosciences 58011482
CD19-BV785 (6D5) Biolegend 115543
CD24-AF700 (M1/69) Biolegend 101836
CD25-BV650 (PC61) Biolegend 102038
CD44-PECy5 (IM7) Biolegend 103010
CD69-BV510 (H1.2F3) Biolegend 104532
CD73-APC (TY/11.8) Biolegend 127210
IFN-γ-PECy7 (XMG1.2) Biolegend 505826
TNF-α-BV650 (MP6-XT22) Biolegend 506333
F4/80-PECy5 (BM8) Biolegend 123114
MHCII-PE/Dazzle™ 594 (M5/114.15.2) Biolegend 121606
CD163-PE (S15049F) Biolegend 156704
CD274-BV605 (10F.9G2) Biolegend 124321

	CD279-APC-R700 (J43) BD Biosciences 565815<br>Ly-6C-BV711 (HK1.4) Biolegend 128037<br>Ly-6G/C-AF594 (RB6-8C5) Biolegend 108448<br>Tim3-APC (B8.2C12) Biolegend 134008<br>CD335(NKp46)-PerCp-eFluor710 eBiosciences 46-3351-82<br>CX3CR1-APC/Fire750 (SA011F11) Biolegend 149039<br>CD86-FITC (GL-1) Biolegend 105006<br>FoxP3-BV421 (MF-14) Biolegend 126419<br>Ly108-BB700 (13G3) BD Biosciences 742272<br>Ki67-PacificBlue (16A8) Biolegend 652422<br>Tox-eFluor660 (TXRX10) eBiosciences 50650282<br>CD103-BV480 (M290) BD Biosciences 566118<br>KLRG1-PECy7 (2F1/KLRG1) Biolegend 138416<br>CD45-APC-Cy7 (30-F11) Biolegend 103116<br>CD8-BUV737 (53-6.7) BD Biosciences 612759<br>CD4-FITC (GK1.5) Biolegend 100406<br>CD279 (PD1)-PE (29F.1A12) Biolegend 135206<br>PanCK-AF532 (AE1/AE3) Novus NBP2-33200AF532<br>CD73-CoraLite594 (1G5G8) Proteintech CL594-67789<br>CD73 Cell signaling 13160s<br>CD3 Viocare medical PP215AA<br>CD8 Cell signaling 70306S<br>Ki67 abcam ab15580<br>PanCK abcam ab7753
Validation	All commercial antibodies used in this work have been by the manufaturer.

Eukaryotic cell lines

Policy information about cell lines and Sex and Gender in Research

Cell line source(s)	MDA-MB231: ATCC<br>MDA-MB468: ATCC<br>4T1: ATCC<br>E0771: ATCC<br>PBMC and purified CD8+ T cell: Zen-Bio
Authentication	The authentication was done by ATCC.
Mycoplasma contamination	All cell lines were tested negative for mycoplasma contamination.
Commonly misidentified lines<br>(See ICLAC register)	No cell lines used in this work are found in the ILAC register of misidentified lines

Animals and other research organisms

Policy information about studies involving animals; ARRIVE guidelines recommended for reporting animal research, and Sex and Gender in Research

Laboratory animals	For our laboratory animal studies, we used BALB/c mice for the 4T1 syngeneic model and C57BL/6 mice for the E0771 syngeneic model. In both models, 7-week-old female mice were used to ensure consistency and minimize variability. The choice of female mice is based on the disease characteristics relevant to these models. Animal housing areas are on a 12 hour x 12 hour light/dark cycle. Relative humidity is controlled within 40-50%. The temperature was controlled at 72°F with a tolerance of ±1°F from the set point year-round.
Wild animals	No wild animals were used in this study.
Reporting on sex	Only female mice were used in this study due to the nature of the disease.
Field-collected samples	No field-collected samples in this study.
Ethics oversight	All animal experiments were approved by the Institutional Animal Care and Use Committee (IACUC) of Emory University and Northwestern University.

Note that full information on the approval of the study protocol must also be provided in the manuscript.

Plants

Seed stocks	N/A
Novel plant genotypes	N/A
Authentication	N/A

Flow Cytometry

Plots

Confirm that:

☒ The axis labels state the marker and fluorochrome used (e.g. CD4-FITC).

☒ The axis scales are clearly visible. Include numbers along axes only for bottom left plot of group (a 'group' is an analysis of identical markers).

☒ All plots are contour plots with outliers or pseudocolor plots.

☒ A numerical value for number of cells or percentage (with statistics) is provided.

Methodology

Sample preparation	For in vitro experiments, cells were detached from 60 mm dishes, washed, and resuspended in PBS before being subjected to flow analysis. For in vivo experiments, tumors were collected, washed, treated with trypsin, and processed into a single-cell suspension before being subjected to flow analysis.
Instrument	BDTM FACSCanto II or CytekTM Aurora
Software	BD FACSDiva software was used for data collection, and FlowJo v10 software was used for analysis.
Cell population abundance	Gating strategies were applied to exclude doublets, dead cells, and debris. The abundance of each cell population was quantified as a percentage of total viable cells within the defined gate using FlowJo v10 software.
Gating strategy	Gating strategies were applied to exclude doublets, dead cells, and debris. The abundance of each cell population was quantified as a percentage of total viable cells within the defined gate using FlowJo v10 software.<br>Initial Gate: FSC vs. SSC (to select live cells and exclude debris).<br>Viability Gate: Exclude dead cells based on live/dead stain.<br>Single-Cell Gate: FSC-A vs. FSC-H (to eliminate doublets).<br>Leukocyte Gate: CD45+ cells.<br>T Cell Gate: CD3+ cells, then subgate for CD4+ and CD8+ T cells.<br>B cell gate: CD3- CD19+ or CD3- CD20+ cells;<br>Myeloid Cell Gate: CD3- CD11b+ cells<br>Dendritic cell Gate: CD3- CD11c+MHC-II+<br>Macrophage cell Gate: CD3- F4/80+ Gr1-<br>MDSC and NK Cell Gates: Based on CD11b, Gr1, CD49b, or NK1.1 expression

☒ Tick this box to confirm that a figure exemplifying the gating strategy is provided in the Supplementary Information.
